# 3D-3D topotactic transformation in aluminophosphate molecular sieves and its implication in new zeolite structure generation

**DOI:** 10.1038/s41467-020-17586-7

**Published:** 2020-07-28

**Authors:** Zhehao Huang, Seungwan Seo, Jiho Shin, Bin Wang, Robert G. Bell, Suk Bong Hong, Xiaodong Zou

**Affiliations:** 1 Bezerlii Center EXSELENT on Porous Materials, Department of Materials and Environmental Chemistry, Stockholm University, SE-106 91 Stockholm, Sweden; 2 Center for Ordered Nanoporous Materials Synthesis, Division of Environmental Science and Engineering, POSTECH, Pohang, 37673 Korea; 3 Research Center for Convergent Chemical Process, Korea Research Institute of Chemical Technology, Daejeon, 34114 Korea; 4 Department of Chemistry, University College London, 20 Gordon Street, London, WC1H 0AJ UK

**Keywords:** Inorganic chemistry, Materials chemistry, Physical chemistry

## Abstract

Zeolites have unique pore structures of molecular dimensions and tunable compositions, making them ideal for shape selective catalysis and separation. However, targeted synthesis of zeolites with new pore structures and compositions remains a key challenge. Here, we propose an approach based on a unique 3D-3D topotactic transformation, which takes advantage of weak bonding in zeolites. This is inspired by the structure transformation of PST-5, a new aluminophosphate molecular sieve, to PST-6 by calcination. The structure of nano-sized PST-5 crystals is determined by 3D electron diffraction. We find that the 3D-3D topotactic transformation involves two types of building units where penta- or hexa-coordinated Al is present. We apply this approach to several other zeolite systems and predict a series of new zeolite structures that would be synthetically feasible. This method provides a concept for the synthesis of targeted zeolites, especially those which may not be feasible by conventional methods.

## Abstract (web summary)

Zeolites have pore structures that are attractive for shape-selective catalysis and separation, but targeted synthesis is challenging. Here, the authors propose using a 3D-3D topotactic transformation to synthesize targeted zeolites, including some that may be not feasible with conventional methods.

## Introduction

Zeolites and related microporous materials have been widely used as selective catalysts, absorbents, and ion-exchangers^1–4^. Recently zeolites have gained interest as catalysts for biomass conversion^5–8^ and automotive exhaust remediation^9,10^. The unique properties of zeolites are associated with the well-defined pores and channels of molecular dimensions. Because of the close structure-property relationship, numerous efforts have been made to synthesize zeolitic materials with new pore structures and compositions and subsequently explore their properties and applications. Currently, only 252 zeolite framework topologies have been approved by the International Zeolite Association^11^, even though millions of possible zeolite topologies have been identified by computational methods^12,13^. The huge gap between experimental output and theoretical prediction indicates that our understanding of zeolite crystallization is still limited. It is, therefore, important to push the field by developing rational strategies to synthesize new zeolites. Besides the conventional hydro-/solvothermal synthesis, several new synthetic strategies have been proposed for targeted zeolite synthesis. These include bottom-up approaches that use, for example, pre-designed organic structure-directing agents (OSDAs)^14–17^ and promotion of specific cavities as in the synthesis of embedded isoreticular zeolites^18,19^, and top-down approaches, i.e., topotactic transformations. The most common top-down approach to synthesize new zeolites has been two-dimensional–three-dimensional (2D–3D) layer condensation from a layered precursor^20–23^. Recently, a new approach of using known zeolites as precursors to synthesize new zeolites via 3D–2D–3D topotactic transformation has gained significant attention^24–27^. The most successful example of these methods is the assembly–disassembly–organization–reassembly (ADOR) synthesis route. The unique concept of the ADOR process is to exploit chemically selective weak T–O bonds (T = Al, Si, Ge, etc.), e.g., Ge–O bonds in germanosilicates, in known zeolites and subsequently promote the formation of new T–O bonds. The ADOR strategy provides a synthetic tool for the generation of zeolites with novel topologies and compositions that cannot be synthesized by conventional hydrothermal methods. To the best of our knowledge, these targeted synthesis strategies have only been demonstrated on silica-based zeolite syntheses (e.g., silicates, aluminosilicates and germanosilicates), but never on phosphate-based frameworks.

Aluminophosphates (known as AlPO_4_-*n*), first synthesized by Union Carbide^28^, are an important class of zeolitic molecular sieves with wide applications. AlPO_4_ molecular sieves have a unique feature, which allows substitution of the Al or P by main block elements (e.g., Si) or transition metals to engender specific properties in the material^29,30^. For example, silicoaluminophosphate SAPO-34 (framework type CHA) is one of the most widely used solid catalysts for biomass conversion, with a global market of tens of billions of US dollars per year. Currently, only 20% of the zeolite framework types can be synthesized as AlPO_4_ phases^11^, and examples of rational synthesis of AlPO_4_ materials are very rare^31^. In AlPO_4_ frameworks, the AlO_4_ and PO_4_ tetrahedra always alternate according to Löwenstein’s rule^32^. Nevertheless, Al can adopt penta-coordination with a pair of Al atoms bridged by a hydroxyl group as found in AlPO_4_-21 (AWO)^33^ and hexa-coordination with two additional water molecules, for example, in AlPO_4_-C (APC)^34^. Upon the loss of water molecules by heating, AlPO_4_-21 and AlPO_4_-C transform into new fully tetrahedrally coordinated zeolite frameworks AlPO_4_-25 (ATV) and AlPO_4_-D (APD), respectively. In 2014, we reported the synthesis and structure of a new AlPO_4_ zeolite denoted PST-6^35^. The framework of PST-6 (PSI) has a one-dimensional (1D) pore system containing parallel 8- and 10-ring channels. PST-6 was obtained by calcination of PST-5 at 500 °C, which was synthesized using diethylamine (DEA) as an OSDA.

Both PST-5 and PST-6 are obtained as nano-size plate-like crystals (Supplementary Fig. 1). The structure of PST-6 was successfully solved by combining electron diffraction and powder X-ray diffraction (PXRD). However, the poorer stability of PST-5 under the electron beam prevented its structure determination, and therefore the understanding of its structure transformation to PST-6. Solid-state nuclear magnetic resonance (NMR) spectroscopy indicated the presence of bridging hydroxyl groups in PST-5.

Recent developments of fast 3D electron diffraction (3DED) or microcrystal electron diffraction (MicroED) data collection techniques (<5 min) using continuous rotation (denoted *c*RED here) have made it possible for studying beam sensitive nano- and micrometer-sized crystals^18,36–40^. Here, we demonstrate the ab initio structure determination of PST-5 using *c*RED data collected under low-dose condition. By detailed investigations of the structure transformation of PST-5 to PST-6, we propose an approach to generate novel zeolite structures from known zeolites via 3D–3D topotactic transformation. The transformation is initiated via the weak Al–O bonding in penta-coordinated AlO_4_(OH) pairs or hexa-coordinated AlO_4_(OH)_2_ units. By adopting this approach, we have, in addition, predicted a series of targeted hypothetical zeolite structures.

## Results

### Structure of PST-5

**Fig. 1:**
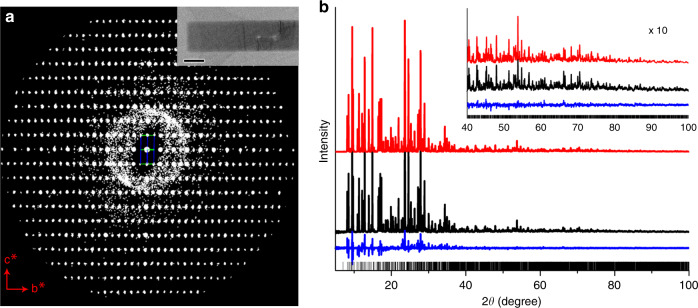
Reconstructed 3D reciprocal lattice and Rietveld refinement. **a** Reconstructed three-dimensional reciprocal lattice of PST-5 from the continuous rotation electron diffraction (*c*RED) data viewed along the [100] direction. Inset is the crystal of PST-5 from which the *c*RED data was collected, scale bar = 500 nm. **b** Powder X-ray diffraction (PXRD) profiles of Rietveld refinement for PST-5 (*λ* = 1.5474 Å): observed, calculated and difference profiles are shown in red, black, and blue, respectively. The bars represent the positions of allowed reflections.

**Fig. 2:**
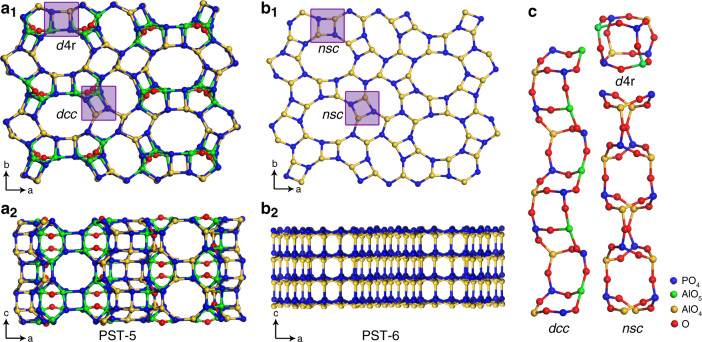
Comparison of the structures of PST-5 and PST-6. Structural models of PST-5 (**a**_**1**_) and PST-6 (**b**_**1**_) viewed along the *c*-axis. Both structures are built from the same building layer containing 4-, 6-, 8-, and 10-rings (if the OH groups, shown in red are not considered). The difference is the orientations of the (Al,P)O_4_ tetrahedra in the two structures. The red atoms in (**a**_**1**_) correspond to the OH groups bridging two Al atoms in PST-5. These Al atoms are five-coordinated. Structural models of PST-5 (**a**_**2**_) and PST-6 (**b**_**2**_) viewed along the *b*-axis, showing the transforms of double-crankshaft chains (*dcc*) and double-4-rings (*d*4*r*) in PST-5 (**a**_**1**_) to narsarsukite-type chains (*nsc*) in PST-6 (indicated by purple boxes). **c** Building units in PST-5 (*dcc* and *d*4*r*) and PST-6 (*nsc*).

A high-resolution *c*RED dataset of PST-5 (with a resolution better than 0.85 Å) was collected from a sub-micrometer-sized crystal using low dose and fast data collection (in total 15 e Å^−2^ and 3 min) on a JEOL JEM2100 transmission electron microscope (Fig. 1a and Supplementary Fig. 2). PST-5 crystallizes in an orthorhombic space group *Pb*2*b* (No. 27), with the unit cell parameters *a* = 36.5956(2) Å, *b* = 21.80273(9) Å, *c* = 10.26929(4) Å (Supplementary Tables 1 and 2). Its framework structure was first solved ab initio by direct methods. To confirm the model and obtain more accurate positions and atomic displace parameters (ADPs) of individual atoms, the model was further refined against the *c*RED data (Supplementary Table 2). Details of structure determination are given in the Supplementary Information. The framework of PST-5 exhibits a totally new topology (Supplementary Figs. 4 and 5, see Supplementary Information for more details) with a fully four-connected 3D net and a 2D pore system containing parallel 8-ring (5.5 × 2.6 Å) and 10-ring (5.8 × 4.4 Å) channels along the ***c***-axis, and another 8-ring (3.3 × 3.3 Å) channel along the ***b***-axis (Fig. 2). PST-5 is one of the most complex zeolite structures, with 18 Al, 18 P, and 76 O atoms in the asymmetric unit. Four of the O atoms belong to hydroxyl groups and act as bridging atoms to form Al–OH–Al linkages. All 18 P atoms and 10 out of the 18 Al atoms are tetrahedrally coordinated with O atom to form TO_4_ units (T = Al, P). The remaining eight Al atoms have trigonal bipyramidal-coordination and form four symmetry-independent AlO_4_-OH-AlO_4_ pairs in the structure. Each AlO_4_-OH-AlO_4_ pair becomes part of a 3- and a 5-ring (Supplementary Fig. 6). Solid-state NMR spectroscopy confirmed the presence of Al–O–Al linkages (Supplementary Figs. 7–9), and a sharp Infrared (IR) band appearing at 3398 cm^−1^ indicates the presence of bridging OH groups (Supplementary Fig. 10). The structure model obtained by Rietveld refinement against synchrotron PXRD data agrees well with that obtained from *c*RED data, which further confirms the framework structure of PST-5. In addition, the OSDA positions in the channels could be located from both *c*RED and PXRD data, and subsequently refined by Rietveld refinement. The final refined unit cell composition is |(C_2_H_5_)_2_NH_2_^+^)_16_ |[Al_72_P_72_O_288_(OH^−^)_16_] (Fig. 1b, Supplementary Figs. 11 and 12 and Supplementary Table 3, see Supplementary Information for more details). Raman spectrum shows that DEA molecules are protonated during PST-5 crystallization^35^. Therefore, the negative framework charges created by the bridging Al–OH–Al groups are balanced by the protonated form of the occluded OSDA molecules (DEA∙H^+^), as reflected by quite similar numbers of OSDA molecules and bridging O atoms.

### 3D–3D topotactic transformation of PST-5 to PST-6

In situ PXRD patterns indicate that the structural transformation of PST-5 to PST-6 starts at 200 °C, and finishes at 500 °C (Supplementary Fig. 13), which is accompanied by notable changes in the PXRD pattern. The observed pattern after calcination of PST-5 at 500 °C agrees very well with the simulated pattern from the PST-6 model, confirming the calcined PST-5 (i.e., PST-6) has the PSI framework (Supplementary Fig. 14). In situ IR studies show that dehydroxylation of bridging OH groups in PST-5 starts above 200 °C and finishes at 300 °C (Supplementary Fig. 10, see SI for more details). Both structures are built from a similar building layer containing 4-, 6-, 8-, and 10-rings if not considering the OH groups (Fig. 2a_1_, b_1_ and Supplementary Fig. 15). Interestingly, the transformation of PST-5 to PST-6 consists not only in the loss of the hydroxyl groups, as observed in the transformation of PST-13 to PST-14^41^. Additionally, it involves T–O bond-breaking and reforming, which leads to totally different connections of the building layers in the two structures (Fig. 2a_2_, b_2_) and thereby different pore systems; 1D for PST-6, in contrast to 2D for PST-5.

The mechanism for the 3D–3D topotactic transformation of PST-5 to PST-6 is investigated based on their structure relationships, as well as molecular dynamics simulations. PST-5 and PST-6 show large structure similarities when viewed along the ***c***-axis (Fig. 2a_1_, b_1_). The framework of PST-5 consists mainly of two types of building units; double-crankshaft chain (*dcc*) and double-4-ring (*d*4*r*), both contain 4-rings, as shown in Fig. 2c and highlighted in Fig. 2a_1_. The framework of PST-6 consists mainly of one type of building unit; a narsarsukite-type chain (*nsc*), as shown in Fig. 2c and marked in Fig. 2b_1_. The transformation of PST-5 to PST-6 can be described by the *dcc*-*nsc* and *d*4*r*-*nsc* transformations. It is worth noting that the *dcc*-*nsc* transformation was also observed in AlPO_4_-21 to AlPO_4_-25^33^, and AlPO_4_-C to AlPO_4_-D^34^. However, the *d*4*r*-*nsc* transformation has not been reported before.

The key to the chain transformation is the penta-coordinated Al atoms, where Al–O bond lengths (on average 1.83(9) Å) are longer than those of tetra-coordinated Al atoms (on average 1.72(1) Å). The longer bond lengths indicate weaker bonds, which are more easily broken. Therefore, the built-in weakness associated with penta-coordinated Al atoms in the structure may be utilized to initiate the topotactic transformation. Upon thermal treatment, the bridging hydroxyl groups are removed through a dehydroxylation to form the fully four-connected framework PST-6 (Fig. 2 and Supplementary Fig. 6).

**Fig. 3:**
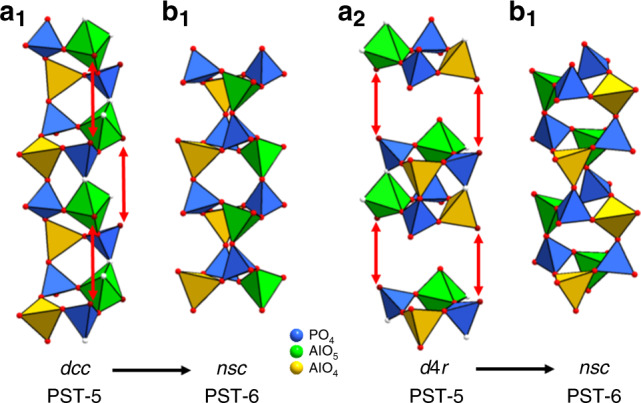
Chain transformations between PST-5 and PST-6. **a**_**1**_ Double-crankshaft chain (*dcc*) and **a**_**2**_ double four-ring (*d*4*r*) unit in PST-5, and **b**_**1**_ and **b**_**2**_ narsarsukite chain (*nsc*) in PST-6. The removal of bridging hydroxyl groups associated with the *dcc* and *d*4*r* initiates the geometry changes of penta-coordinated Al atoms, resulting in cleavage of Al–O–P bonds (corresponding O atoms are shown in white) followed by inversion and reconnection of the tetrahedra (indicated by arrows). Both *dcc* and *d*4*r* transform to *nsc* in PST-6. Green, penta-coordinated (and penta-coordinated derived) AlO_4_ tetrahedra; yellow, tetra-coordinated AlO_4_; blue, PO_4_. Oxygen atoms associated with OH or bond-breaking in the chains are shown in white and the rest O in red.

By detailed inspection of the structural relationships between PST-5 and PST-6, the geometry changes of penta-coordinated Al atoms initiate a domino geometrical inversion of nearby PO_4_ tetrahedra. Upon heating, the penta-coordinated Al atoms lose the bridging hydroxyl groups, which also leads to cleavage of the Al–O–P bond to the adjacent PO_4_ tetrahedra. The AlO_4_ and PO_4_ tetrahedral pairs with resulting broken bonds then invert their orientations and reconnect to the next nearest pairs of AlO_4_ and PO_4_ tetrahedra (Fig. 3, indicated by arrows). As a result, the double-crankshaft chains (*dcc*) in PST-5 transforms to the narsarsukite-type chains (*nsc*) in PST-6, which is composed of one chain with tetra-coordinated Al atoms, and another chain originating from penta-coordinated Al atoms (Fig. 3a_1_, b_1_). The inversion further extends to neighboring atoms, which results in a domino-like alteration of the tetrahedral conformation. There are two symmetry-independent *dcc* chains in PST-5 with different orientations. After being transformed to PST-6, they orient in the same way. The reconstructed PST-6 (PSI) material was found to contain stacking faults^35^, due to changes in both orientation and tetrahedral conformation. There are eight unique penta-coordinated Al atoms that can initiate the topotactic transformation. When the transformation starts at different positions, it will also generate disorder after the reconstruction.

To study the possible mechanisms for the transformation of PST-5 to PST-6, periodic ab initio molecular dynamics simulations were carried out on a model PST-5 system. In nearly every case, one bond from penta-coordinated Al was broken, forming tetra-coordinated Al and corresponding P-OH groups (Supplementary Figs. 16 and 17). In one case, the Al–O–P linkage was broken within a 3- (–Al–O–P–Al–OH–) ring, but otherwise the framework interruption occurred between the penta-coordinated Al and neighboring P atoms, which were part of a double-crankshaft chain (*dcc*) or a double-4-ring (*d*4*r*) structural unit but not in a 3-ring. The simulations clearly confirm that weaker bonds in the PST-5 framework are associated with penta-coordinated Al atoms and are labile in the presence of an extra-framework proton donor. The initial broken bonds are those in the *dcc* and *d*4*r*, largely the same as those implicated in the transformation of PST-5 to PST-6 at the experimental annealing temperature. Coupled with the topological strain inherent within the *dcc* and *d*4*r* due to their fused 4-ring motifs, the penta-coordinated Al atoms are thus critical in introducing reactivity into the framework. It should be noted that the molecular dynamics simulations of an idealized, fully four-connected, AlPO_4_ PST-5 framework, without penta-coordinated Al atoms and bridging OH groups, showed no cleavage of any framework bonds over the same timescale at 500 °C.

### New zeolite generation

**Fig. 4:**
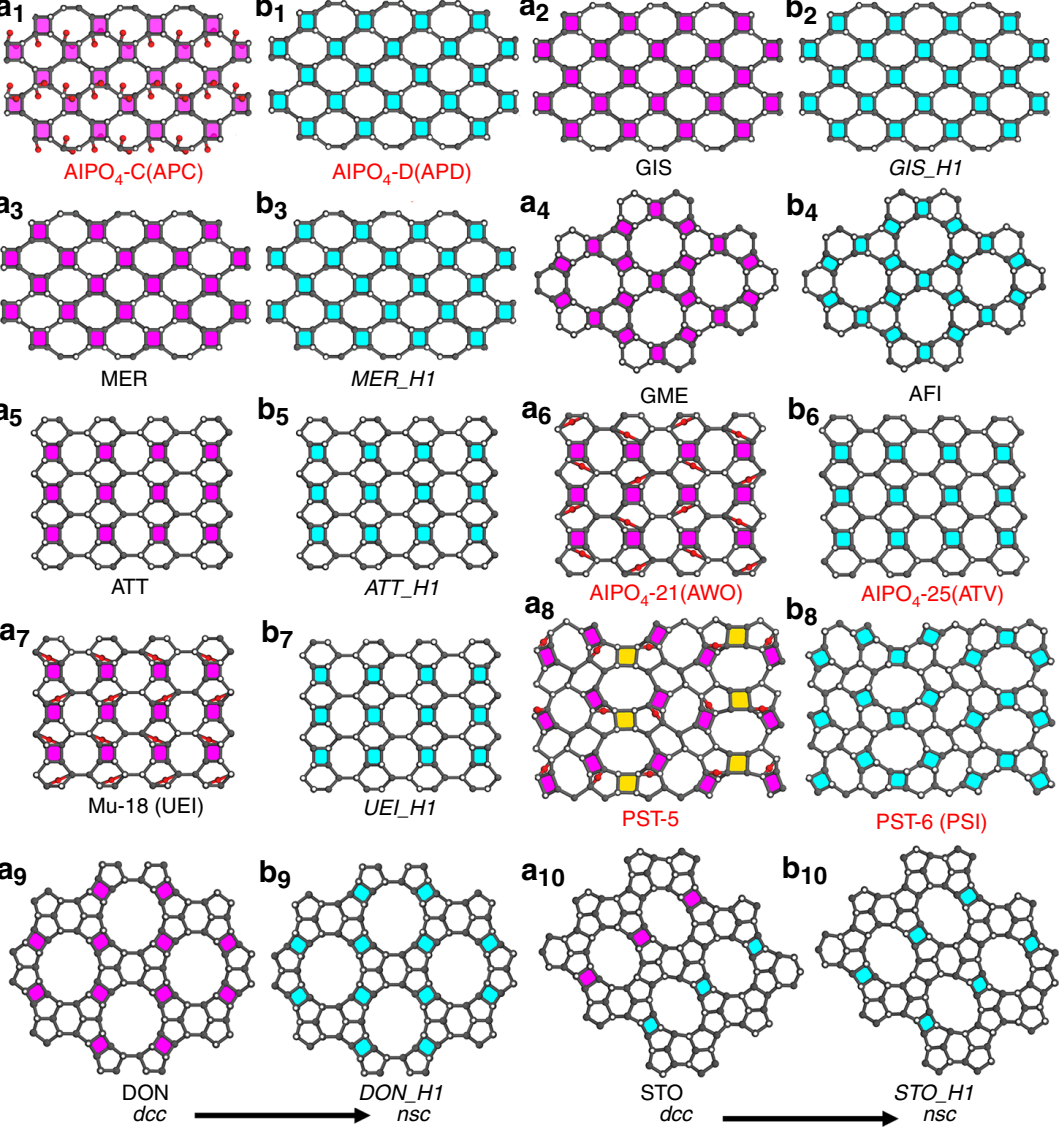
Illustration of observed and predicted 3D–3D topotactic transformations. **a**_**1**_–**a**_**10**_ Projections of the APC, GIS, MER, GME, ATT, AWO, UEI, PST-5, DON, and STO frameworks. All of them contain double-crankshaft chain (*dcc*). Among them, APC, AWO, UEI, and PST-5 contain penta- or hexa-coordinated atoms. **b**_**1**_, **b**_**6**_, and **b**_**8**_ Projections of APD, ATV, and PSI (PST-6) frameworks after topotactic transformation by changing *dcc* to narsarsukite chain (*nsc*). They have the same framework projections as APC, AWO, and PST-5, respectively, but differ in the 3D connectivity along the projections. **b**_**2**_–**b**_**5**_, **b**_**7**_, **b**_**9**_, and **b**_**10**_ Selected hypothetical structures of *GIS_H1*, *MER_H1*, *ATT_H1*, *UEI_H1*, *DON_H1*, and *STO_H1* generated by replacing *dcc* with *nsc*. Magenta: *dcc*; cyan: *nsc*; yellow: double four-ring (*d*4*r*). The solid and hollow circles indicate vertices that connect upwards and downwards, respectively.

Inspired by the 3D–3D topotatic transformation in PST-5/PST-6, we investigated the existing structures in the Zeolite Structure Database^11^. 12 framework types are found to contain infinite *dcc*, and they can be divided into three groups based on the structural complexity (Table 1): I) built solely from *dcc* that are connected directly (APC, GIS, MER, PHI, SIV, GME, Supplementary Fig. 18); II) built from *dcc* that are bridged by isolated 4-rings (ATT, AWO, UEI, Supplementary Figs. 19 and 20); III) built by *dcc* and other building units (PST-5, DON, STO, Supplementary Fig. 21). Interestingly, regardless of the complexity, the *dcc*-*nsc* structural transformation has been observed in all groups, from AlPO_4_-C (APC) to AlPO_4_-D (APD) in group I, AlPO_4_-21 (AWO) to AlPO_4_-25 (ATV) in group II, and PST-5 to PST-6 (PSI) in group III (Table 1 and Supplementary Table 4). The three parent frameworks all contain *dcc* in which penta- (AlPO_4_-21 and PST-5) or hexa- (AlPO_4_-C) coordinated Al atoms are present. By applying similar *dcc* to *nsc* transformations observed in the three known zeolite pairs to other *dcc*-containing zeolites, we generated a series of novel hypothetical *nsc*-containing zeolite structures, as shown in Fig. 4, Supplementary Figs. 18–22, Table 1, and Supplementary Table 5 (see Supplementary Information for more details)^42^. Notably, the same parent zeolite can generate more than one hypothetical zeolites (e.g., UEI to *UEI_H1-5*), and the same zeolite can be generated from different parent zeolites (e.g., *GIS_H1* from both GIS and SIV). The transformation can also generate new channel dimensionality (Supplementary Fig. 18 and Table 1). All these hypothetical *nsc*-containing zeolites fulfill the criteria for zeolite frameworks realizable for target synthesis (Supplementary Table 6)^43^. Most of them were calculated to have much lower framework energies than the parent *dcc*-containing zeolites. It is worth mentioning that because each TO_4_ tetrahedral unit not belonging to *dcc* can have two possible connectivities, either upwards or downwards, the number of hypothetical zeolites generated via this approach can be enormous for zeolites in group II and III (see Supplementary Information for more details).

**Table 1:** Observed (in bold) and predicted 3D–3D topotactic transformations of zeolites through chain transformation from *dcc* to *nsc* and *d*4*r* to *nsc*.

FTC^a^ (parent)	*E*_F_^b^	Channel dimensionality	FTC (transformed)	*E*_F_^b^	Channel dimensionality
Group I: *dcc* → * nsc*					
**APC**	**14.4**	**2D**	**APD**	**12.3**	**2D**
GIS	15.0	3D	*GIS_H1*	10.3	1D
SIV	15.3	^3D^	*GIS_H1*	10.3	1D
MER	16.2	3D	*MER_H1*	12.3	2D
PHI	15.7	3D	*MER_H1*	12.3	2D
GME	16.5	3D	AFI	11.0	1D
Group II: *dcc* + isolated 4-rings → *nsc* + *scc*^c^					
ATT	15.1	2D	*ATT_H1*	10.8	2D
**AWO**	**14.0**	2D	**ATV**	**8.9**	**1D**
			*AWO_H1*	12.2	1D
			*AWO_H2*	14.7	1D
UEI	14.2	2D	*UEI_H1*	11.2	1D
			*UEI_H2*	11.3	1D
			*UEI_H3*	11.0	1D
			*UEI_H4*	11.3	1D
			*UEI_H5*	11.5	1D
Group III: *dcc* + other building units → * nsc* + other building units					
**PST-5**	**15.9**	**2D**	**PSI**	**9.2**	**1D**
DON	10.2	1D	*DON_H1*	13.2	1D
STO	9.7	1D	*STO_H1*	10.6	1D

^a^Framework Type Code approved by International Zeolite Association.

^b^Framework energy (kJ (mol Si) ^– 1^) relative to α-quartz.

^c^Single crankshaft chain.

We have demonstrated that many hypothetical zeolites can be predicted from known zeolite frameworks based on the *dcc*-*nsc* transformation. The 3D–3D topotactic transformation would provide a simple approach to generate novel zeolite frameworks, which may be inaccessible by conventional hydrothermal/solvothermal synthesis. We have also shown by molecular dynamics simulations that the weakened bonding, such as penta- or hexa-coordinated Al atoms, is crucial to initiate the 3D–3D topotactic transformation. Therefore, to synthesize new structures by this approach, it is important to introduce penta- or hexa-coordinated Al atoms in *dcc*-containing zeolite frameworks, and additional exploratory work is needed. Previous studies showed that certain types of OSDAs, like easily protonatable amines, could promote the formation of bridging Al–OH–Al bonds in AlPO_4_ molecular sieves, and penta- or hexa-coordinated Al atoms have been found in c.a. 25% of these materials, including AlPO_4_-17, AlPO_4_-41, AlPO_4_-EN3, AlPO-CJB1, VPI-5, IST-1, PST-13, etc.^41,44–50^. The 3D–3D topotactic transformation may also be applicable to other zeolite compositions. For example, the gallophosphate Mu-18 (UEI) contains both *dcc* and penta-coordinated atoms (Fig. 4a_7_)^51^. We anticipate that with the discovery of many new zeolites, the approach described here will be more feasible.

## Discussion

We have solved the structure of PST-5, a new 2D medium-pore AlPO_4_ molecular sieve, using continuous rotation electron diffraction (*c*RED). Based on the investigations of the *dcc* to *nsc* transformations observed in PST-5/PST-6, AlPO_4_-C/AlPO_4_-D, and AlPO_4_-21/AlPO_4_-25 pairs, we propose an approach to generate novel zeolite structures, which enriches the structural diversity. The 3D–3D topotactic transformation relies on the chemical weakness in the *dcc*. PST-5 transforms *dcc* and *d*4*r* to *nsc*, changes its overall interlayer connectivities and finally generates PST-6. The penta-coordinated Al atoms, as observed in PST-5, could be built into a precursor material by using pre-designed OSDAs, which balance the framework energy. This approach via the 3D–3D topotactic transformation can be considered complementary to the existing strategies of synthesizing novel zeolite structures. By understanding the 3D–3D topotactic transformation, we anticipate that our approach can be generalized and provide new insights into the discovery of novel zeolitic materials, which may enable a completely new range of zeolite frameworks to be accessible and offer opportunities in technological applications.

## Methods

### Synthesis of PST-5

The reagents used for the synthesis of PST-5 included diethylamine (DEA, 99.5+%, Aldrich), aluminum isopropxide (98+%, Aldrich), phosphoric acid (85%, Merck), and deionized water. PST-5 was synthesized from an AlPO_4_ gel with the composition of 2.0DEA·1.0Al_2_O_3_·1.0P_2_O_5_·40H_2_O, where DEA is diethylamine. In a typical synthesis of PST-5, 3.84 g of *o*-H_3_PO_4_ (85%, Merck) was diluted in 5.63 g of H_2_O. This solution was added dropwise to a slurry of 6.95 g of aluminum isopropoxide (>98%, Aldrich) in 5.64 g of H_2_O. After stirring for 1 h, 2.45 g of DEA (99.5%, Aldrich) was added to the above synthesis mixture, which was stirred overnight at room temperature. Afterwards, the mixture was charged into a Teflon-lined 23-mL autoclave and heated under static conditions at 200 °C for 5 days. The resulting white powder was recovered by filtration, washed repeatedly with water and then dried overnight at room temperature.

### Topotactic transformation of PST-5

The topotactic transformation of PST-5 to PST-6 was conducted by calcination of PST-5 at 550 °C for 8 h under air flow.

### *c*RED data collection

PST-5 powder in its as-made form was dispersed in ethanol and a droplet of the suspension was transferred onto a carbon-coated copper grid and dried in air. *c*RED data was acquired in selected-area mode with a goniometer tilt speed of 0.45° s^−1^ and an exposure time of 0.4 s per frame (Supplementary Table 2). The total data collection time was <3 min in order to minimize the beam damage and maximize the data quality. The total tilt range was 92.6°.

## Supplementary information

Supplementary Information

Peer Review File

## Data Availability

The crystallographic information files for PST-5 refined against the cRED and PXRD data have been deposited at the Cambridge Crystallographic Data Center (CCDC, free for charge at https://www.ccdc.cam.ac.uk) under deposition number CCDC 1948775 and 1944918, respectively. The cif containing all the hypothetical structures and Supplementary information are available in the online version of the paper. Reprints and permissions information is available online at www.nature.com/reprints. Correspondence and requests for materials should be addressed to S.B.H. (sbhong@postech.ac.kr) and X.Z. (xzou@mmk.su.se).
