# Supplementary material for: 3D-3D topotactic transformation in aluminophosphate molecular sieves and its implication in new zeolite structure generation
Source: Nat Commun. 2020 Jul 28;11:3762. doi: 10.1038/s41467-020-17586-7 (PMC7387333; doi:10.1038/s41467-020-17586-7)
Supplement: Supplementary file 1 — Supplementary Information [file 41467_2020_17586_MOESM1_ESM.pdf]

Supplementary Information
for
3D-3D Topotactic Transformation in Aluminophosphate Molecular Sieves and Its Implication in New Zeolite Structure Generation

Zhehao Huang,[1†] Seungwan Seo,[2†] Jiho Shin,[3] Bin Wang,[1] Robert G. Bell,[4] Suk Bong Hong[2*] and Xiaodong Zou[1*]

[1]Bezerlii Center EXSELENT on Porous Materials, Department of Materials and Environmental Chemistry, Stockholm University, SE-106 91 Stockholm, Sweden

[2]Center for Ordered Nanoporous Materials Synthesis, Division of Environmental Science and Engineering, POSTECH, Pohang 37673, Korea

[3]Research Center for Convergent Chemical Process, Korea Research Institute of Chemical Technology, Daejeon 34114, Korea

[4]Department of Chemistry, University College London, 20 Gordon St., London WC1H 0AJ, UK

† These authors contributed equally to this work.

Corresponding Authors: sbhong@postech.ac.kr; xzou@mmk.su.se

Supplementary Notes

General Characterization:

In-situ variable-temperature X-ray diffraction (XRD) experiments were performed in Bragg-Brentano geometry using a PANalytical X′Pert diffractometer (Cu Kα radiation) equipped with an Edmund Bühler HDK 1.4 high temperature attachment. Variable-temperature IR spectra were measured on a Nicolet 6700 FT-IR spectrometer using self-supporting zeolite wafers of 15 mg (1.3 cm diameter). Prior to IR measurements, the zeolite wafers were pretreated under vacuum at 120 °C for 6 h inside a homebuilt IR cell with CaF_2 windows. Then, the spectra were recorded under vacuum (1×10^{-4} Pa) from room temperature to 400 °C. Elemental analysis for framework elements was carried out by a Jarrell-Ash Polyscan 61E inductively coupled plasma spectrometer in combination with a Perkin-Elmer 5000 atomic absorption spectrophotometer. The C, H and N contents of the samples were analyzed by using a Carlo Erba 1106 elemental organic analyzer. Thermogravimetric and differential thermal analyses (TGA/DTA) were performed in air on a TA Instruments SDT 2960 thermal analyzer at a heating rate of 10 °C min^{-1}.

^{27}Al MAS NMR spectra were recorded on Varian *InfinityPlus* 500 and Inova 800 spectrometers operating at 11.7 and 18.8 T, corresponding to ^{27}Al Larmor frequencies of 130.1 and 208.4 MHz, respectively. The spectra were obtained with a π/12 rad pulse length and a recycle delay of about 0.2 s. ^{31}P MAS NMR spectra were measured on the same spectrometers, corresponding to ^{31}P Larmor frequencies of 202.1 and 324.0 MHz, respectively. The spectra were obtained with a π/4 rad pulse length and a recycle delay of 300 s. The samples were loaded in MAS rotors and spun at the magic angle at rates of 10 - 16 kHz and all measurements were done at room temperature. The ^{27}Al and ^{31}P chemical shifts are referenced with respect to external solutions of $Al(H_2O)_6^{3+}$ (δ_{Al} = 0.0 ppm) and 85% H_3PO_4 (δ_P = 0.0 ppm), respectively. ^{27}Al and ^{31}P spectra were recorded on the hydrated PST-5, PST-6 (i.e., calcined PST-5), vacuum dried PST-6 at 150 °C for 10 h and dried-rehydrated PST-6. Rehydration of the dried PST-6 material was done by exposing the sample to a 100% relative humidity environment overnight. ^{27}Al multiple-quantum (MQ) MAS NMR and ^{27}Al → ^{31}P MQHETCOR spectra were recorded at 11.7 T using a Varian triple-resonance 4-mm T3 TR probe. The MQMAS[1,2] pulse sequence used was 3QMAS with *z*-filter. MQHETCOR data were recorded with a pulse sequence[3] that combines the MQMAS experiment with the CPHETCOR one to generate heteronuclear correlation data with isotropic resolution in both dimensions.

Structural Analysis:

The traditional methods of structure determination, such as single crystal XRD and powder XRD, were unsuccessful in obtaining the detailed structure of PST-5 and in revealing the mechanism of the topotactic transformation. The prime reasons lay not only in the nano-sized nature of the crystallites but also in the structural complexity and beam sensitivity of PST-5. Hence, continuous rotation electron diffraction (*c*RED) was applied, which allows accurate determination of the crystal structure of such a complex material. In addition to the benefits of electron crystallography, which enables structure determination of very small (< 50 nm) crystals, the *c*RED method allows fast data collection (< 1 min) which thus minimizes any beam damage, as well as extracting more accurate structural information.[4,5] ITQ-58 was the first zeolite solved using this method.[6]

Importantly, there is an indication that PST-5 contains penta-coordinated Al atoms. Such atoms could bind to hydroxyl groups or water molecules, which could be easily removed under high vacuum conditions in the TEM. Therefore, to prevent the removal of these groups, the TEM grid with the PST-5 crystals was cooled down to -178 ºC using a cooling holder during the study.

The *c*RED dataset was collected from an as-made PST-5 crystal which diffracted to 0.85 Å resolution. The data was processed using the XDS package,[7] which showed that PST-5 is orthorhombic with a primitive unit cell. The completeness is 70.5% and the R_{int} value is 0.179. The unit cell parameters were determined from the *c*RED data, which were further refined to be a = 36.59524(16) Å, b = 21.80267(8) Å and c = 10.269318(35) Å using PXRD. The space group of PST-5 was determined from *c*RED data according to the reflection conditions as 0*kl*: $k = 2n$; *hk*0: $k = 2n$; 0*k*0: $k = 2n$ (Supplementary Fig. 2). There are two possible space groups for PST-5, *Pb*2*b* (No. 27) and *Pbmb* (No. 49). The framework structure of PST-5 was determined by direct methods using the space group *Pbmb* and program Shelx-2014.[8] 34 T-atoms and 71 oxygen atoms were found directly. The remaining 2 T-atoms and 5 O atoms were located from difference Fourier maps. After solving the framework structure, the space group was reduced to *Pb*2*b*, which allows alternating AlO_4 and PO_4 tetrahedra as required for $AlPO_4$ structures. Due to the structural relevance between PST-5 and PST-6, in addition, the non-standard space group was chosen so that the comparison could be more straightforward.

All of the 8 penta-coordinated Al atoms were identified directly from the structure solution, which have trigonal bipyramidal coordination. The remaining 10 Al and 18 P atoms showed clearly tetrahedral-coordination to O atoms. The final refinement was conducted using Shelxl-2014, which converged to R_1 = 0.278. The high R_{int} and R_1 values may be caused by dynamical effects, where calculated kinematical intensities were compared to the experimental dynamical intensities in the *c*RED data. The details of data collection and structure refinement against *c*RED data are summarized in Supplementary Table 2.

The structural model of PST-5 obtained from the *c*RED method was further refined using synchrotron powder X-ray diffraction (PXRD). Synchrotron PXRD data were collected on the 9B beamline of the Pohang Acceleration Laboratory (PAL; Pohang, Korea)

using monochromated X-rays (λ = 1.54740 Å). The detector arm of the vertical scan diffractometer consists of seven sets of soller slits, flat Ge (111) crystal analyzers, anti-scatter baffles and scintillation detectors, with each set separated by 20°. The data were obtained on the sample in flat plate mode, with a step size of 0.01° and overlaps of 2.0° to the next detector bank over the 2θ range 4.0-124.5°. The synchrotron PXRD pattern of PST-5 was indexed as *Pb*2*b*, with a = 36.59524(16) Å, b = 21.80267(8) Å and c = 10.269318(35) Å. The Rietveld refinement was obtained using the GSAS package and EXPGUI graphical interface.[9–11] The background curve was fitted using a Chebyschev polynomial with 36 terms. The Bragg peak profiles were modeled using a pseudo-Voigt (type IV) function.[12] The tetrahedral framework Al−O, P−O, O−O (Al) and O−O (P) distances were soft-restrained to 1.72 Å, 1.52 Å, 2.82 Å and 2.50 Å (σ = 0.01 Å), respectively. The penta-coordinated framework Al−O and O−O (Al) distances were soft-restrained to 1.63−1.95 Å (σ = 0.01 Å) and 2.38−3.88 Å (σ = 0.05 Å), respectively. The restraint weight was gradually decreased during the refinement, lowered to a final weight of 10. DEA, which was used as the organic structure-directing agent (OSDA) for synthesizing PST-5, was applied as a rigid body for its location and orientation determination. The OSDA positions were determined using the parallel tempering method implemented in the FOX program.[13] The result matches well with that determined from the difference Fourier analysis. The rigid bodies were restrained by interatomic distance and angle restraints and were allowed to translate and rotate as a whole. The C−C and C−N distances and C−N−C and N−C−C angles in the organic cations were restrained to 1.44−1.52 Å and 109.35−109.91°, respectively. The isotropic thermal displacement parameters of the framework atoms and organic molecules were constrained in groups for the (Al, P), O and (C, N) atoms, respectively. An overall isotropic atomic displacement parameter has been fixed for all water O atoms. The final R_{wp} and R_p values converged at 0.092 and 0.067, respectively. The data collection and crystallographic parameters for PST-5 are summarized in Supplementary Table 3 and the refined atomic parameters, bond lengths and angles for PST-5 can be found in crystallographic information file. The refined unit cell composition $|(C_4H_{12}NH_2^+)_{16.0}|$ $[Al_{72}P_{72}O_{288}(OH^-)_{16}]$ of PST-5 is in good agreement with that ($|(C_4H_{11}N)_{17.6}(H_2O)_{12.6}|[Al_{72}P_{72}O_{288}(OH)_{16}]$) obtained by a combination of elemental and thermal analysis. Only a small fracture of water molecules could be located from the difference Fourier maps. This is because most water molecules are disordered in the pores.

The variable-temperature *in-situ* IR results (Supplementary Fig. 10) reveal a sharp band around 3400 cm^{-1}, which is assigned to the OH stretching vibration of Al−OH−Al linkages[14–16]. This remains present at temperatures up to 200 °C and disappears at 300 °C, in good agreement to the thermal analysis results (Supplementary Fig. 12) that the bridging OH groups are being removed at that temperature. Variable-temperature *in-situ* powder XRD data (Supplementary Fig. 13) show little change in the structure until the temperature rises to 200 °C, at which the bridging OH groups start to be removed. However, noticeable changes in the position and relative intensity of X-ray diffraction peaks begin to be observable when the temperature reaches 300 °C. For instance, the peaks appearing around

2θ = 14.8 and 24.5°, which correspond to 231 and 060 reflections of PST-5, respectively, become weaker, whereas those around 2θ = 10.4 and 15.9° related to the structure of PST-6 start to appear.

The average distances of tetrahedral P−O and Al−O bonds in the PST-5 framework (1.525 and 1.724 Å, respectively) correspond well with the expected value ranges of typical $AlPO_4$ molecular sieves. However, those of penta-coordinated Al−O bonds (1.836 Å) are longer than the average distance of tetrahedral Al−O bonds (1.724 Å, see crystallographic information file). This is consistent with the average distances (1.835 and 1.884 Å, respectively) of similar bonds in as-made $AlPO_4$-EN3 (AEN) and $AlPO_4$-21 (AWO)[17,18], which also contain penta-coordinated Al−O bonds.

The topotactic transformation involves key connectivity changes of the interlayer that can also be described by the direction of the interlayer linkages. These can be either upwards (U) or downwards (D) to the adjacent 2D nets (Supplementary Fig. 15a, indicated by solid and hollow circles, respectively). The resulting chains and units of the 4-rings in the 3D structure are double-crankshaft chain (*dcc*; interlayer connectivity UUDD), double 4-ring (*d4r*; DDDD) and narsarsukite-type chain (*nsc*; UDUD) as in the boxes I, II and III, respectively (Supplementary Fig. 15). The two position changes of the penta-coordinated Al atom (from trigonal bipyramids to tetrahedra), as well as the P atom (inversion of the tetrahedral conformation), is possibly the reason that disorder is generated in PST-6[19].

Framework Topology:

PST-5 exhibits a new framework topology, as shown in Supplementary Figs. 4 and 5. The structure of PST-5 is composed of six different building units: [4^6], [$3.4^9.5.6^2.8^3$], [$3^2.4^4.5^6.8^2$], [$3.4^2.5^3.6^2.8^2$], [$4^4.8^2.10^2$] and [$3^2.4^8.5^2.8^2.10^2$], the last two contain both 8- and 10-rings (Supplementary Fig. 4). The building units are connected to form a basic layer by sharing the faces (Supplementary Fig. 15a). The layers are connected to the neighboring layers along the ***c***-axis to form the entire 3D framework structure (Supplementary Fig. 15b). Due to the existence of penta-coordinated Al atoms, except [4^6], the rest of the building units have not been reported before and are much more complicated than those in common zeolite structures.

After the topotactic transformation, the framework density increased by ~7 % from PST-5 to PST-6 (Supplementary Table 1). The 2D channel system in PST-5 should result in a better diffusion performance than PST-6. Furthermore, the interlayer distance is shortened by 1.94/2 = 0.97 Å (from 10.27 Å to 8.33 Å, both structures contain two layers). The *a*-parameter is reduced mostly, as a result of the formation of 8-membered channels along *b*-axis[20].

***Ab initio* Molecular Dynamics Simulation:**

Born-Oppenheimer molecular dynamics simulations were carried out with periodic DFT using the CP2K code,[21–23] which utilizes a combined Gaussian-Plane Wave (GPW)

approach[24]. The PBE functional[25] was used together with the DZVP-MOLOPT-SR-GTH basis set[22] for all atoms. Core electrons were represented by the GTH pseudopotentials[25]. The simulations were carried out on a model PST-5 system, in which the extra-framework OSDA and other species were replaced by H_3O^+ ions to charge balance the framework OH. The simulation cell was doubled in the *z* direction and thus included 64 penta-coordinated Al atoms, 32 OH-containing 3-rings and 32 H_3O^+ species. The simulation was run at a temperature of 500 °C for 10 ps and in the NVT ensemble using the Nosé-Hoover thermostat with a timestep of 1 fs. After 10 ps, 31 of the 32 H_3O^+ ions were deprotonated, with 30 framework P-O-Al linkages being broken and one Al-OH-Al linkage. During the simulation, very rapid exchange of protons between the framework and H_3O^+ ions was observed. There was also some distortion to the 3-membered rings visible during the simulation, but after 10 ps, 75% of the 3-membered rings (24 out of 32) remained intact, with Al-O and P-O bond lengths within reasonable tolerances. We were not able to use molecular dynamics to simulate the recrystallisation process. Indeed, longer runs resulted in further amorphisation of the material.

Hypothetical Zeolite Structure Generation:

The hypothetical zeolite structures were firstly generated in Material Studio 7.0 by changing the connectivities of T-atoms. The O atoms were later added geometrically and the structures were optimized using DLS-76,[26] Dreiding potential,[27] (without charges) and then further optimized using the Sanders-Leslie-Catlow (SLC) potential[28] in the GULP program.[29] The topologies were evaluated by using the ToposPro program[30], and duplicated topologies were removed based on the same coordination sequences. The highest symmetries of the models were identified using Materials Studio 7.0. The symmetrized structures were standardized in their corresponding Niggli reduced cells in the VESTA program.[31] To evaluate the feasibility of hypothetical models with $P1$ symmetry, their framework energies relative to α-quartz[32] were calculated using the SLC potential in the GULP program. In addition, the local interatomic distances (LID) criteria were applied.[33]

The generation of hypothetical zeolite structures follows the same principles as those observed (Supplementary Figs. 18-21, Supplementary Table S4). We found the number of possible hypothetical structures increases based on the complexity of the parent framework structures (Table 1 and Supplementary Table 5). Introduction of the *dcc-nsc* transformation on the known zeolites in group I (APC, GIS, MER, PHI, SIV and GME), which is built solely by *dcc*, results in two known zeolite frameworks and two hypothetical structures, as shown in Supplementary Fig. 18. While APC and GME transform to the already known framework APD and AFI, respectively, MER and PHI result in the same hypothetical structure (denoted *MER_H1*) and GIS and SIV yield the same framework (denoted *GIS_H1*). APC, GIS, MER, PHI and SIV all have the same 2D topology in the projection along the chain direction. The different 3D topologies originate from different relative orientations of adjacent *dcc*. While there are four possible orientations for each *dcc*, each *nsc* has only two possible orientations.

The number of hypothetical structures increases drastically when additional TO_4 tetrahedral units are incorporated in the framework. In group II (ATT, AWO and UEI), the additional TO_4 tetrahedral units are isolated 4-rings, which are aligned in columns connecting the *dcc* in parent structures. The 4-ring columns transform to single crankshaft chains (*scc*) that connect *nsc*, as shown in Supplementary Fig. 20. Each *scc* can have two possible orientations. If we keep the orientation relationships of the *dcc* to *nsc* transformation the same as observed in AWO to ATV transformation, the number of possible hypothetical structures are related to the number (n) of *scc* in each unit cell, i.e. 2^n. Supplementary Fig. 19 shows the hypothetical *nsc*–containing structures in group II. The ATT family has only one 4-ring column/*scc* per unit cell. This results in two possible hypothetical structures, which have identical topology (denote *ATT_H1*). The AWO family has two 4-ring columns/*scc* per unit cell, leading to four (2^2) possible structures and three unique topologies; one is known (ATV) and two are new (*AWO_H1* and *AWO_H2*). The UEI family contains four 4-ring columns/*scc* per unit cell, which generates 2^4=16 possibilities, of which five have unique topologies (*UEI_H1-5*). It is worth noting that if the *nsc* are allowed to have different orientations, much more hypothetical structures can be generated.

In group III (PST-5, DON and STO), much higher numbers of additional TO_4 tetrahedral units are present, thus the number of hypothetical structures is expected to be also higher than those in found group II. We therefore give only one example of hypothetical structure for each zeolite framework (*DON_H1* and *STO_H1*), which is deduced by replacing *dcc* with *nsc*, while keeping the rest of the framework connectivities unchanged (Supplementary Fig. 21). The transformation of PST-5 to PST-6 indicates that the 3D-3D topotactic transformation can occur in complicated framework structures as well. As a consequence of different interlayer connectivities (Supplementary Figs. 20 and 22), the hypothetical structures can result in different channel dimensionality compared to their parent frameworks (Table 1).

We also carried out framework energy calculations and evaluations of LID criteria among different hypothetical structures generated from the same parent framework. The calculated framework energies of all these structures are always lower than 30 kJ (mol Si)$^{-1}$ relative to α-quartz, and all of them satisfy the LID criteria of feasible zeolite structures (Supplementary Table 6)[33,34]. This supports the reliability of our new method to generate feasible zeolite structures. With different connectivities of the atoms connecting *nsc*, the framework energy becomes different. As shown in Table 1, all the known 3D-3D topotactic transformations have higher framework energies with *dcc* than those containing *nsc*. The energy differences between a parent structure and a hypothetical structure are in the range of 2.9 - 4.7 kJ (mol Si)$^{-1}$. Given that the energy difference between APC and APD is 2.1 kJ (mol Si)$^{-1}$, those hypothetical structures are likely to be synthesizable. Among the hypothetical structures generated in this study, *AWO_H2*, *DON_H1*, and *STO_H1* were calculated to have higher framework energies.

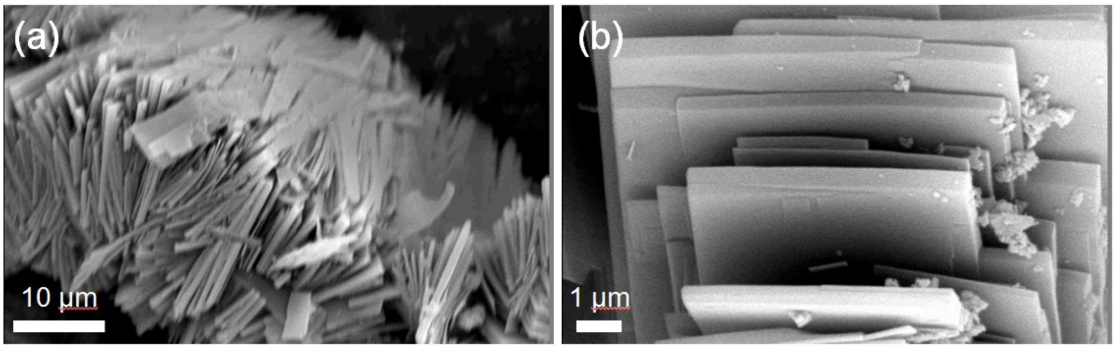

Supplementary Figure 1. SEM images of (a) PST-5 and (b) PST-6.

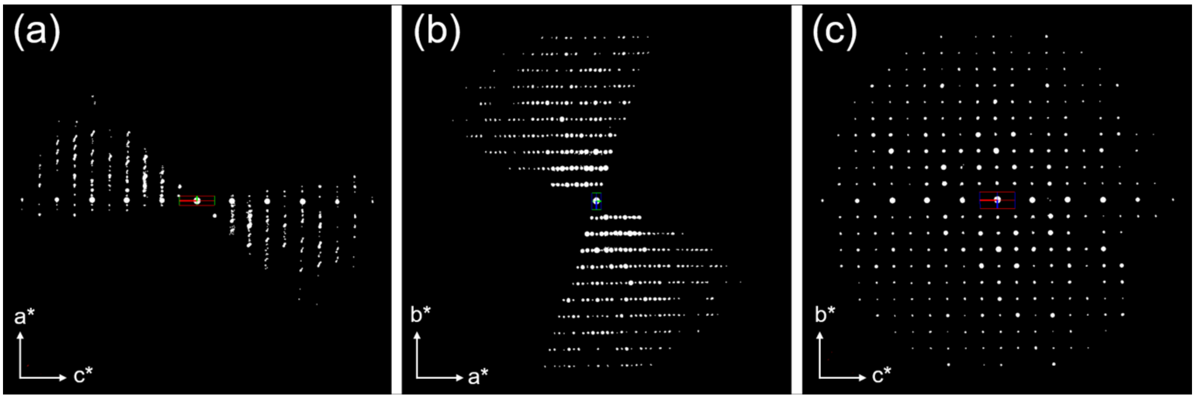

Supplementary Figure 2. 2D slices cut from the reconstructed 3D reciprocal lattice of PST-5. (a) *h*0*l* plane, (b) *hk*0 plane and (c) 0*kl* plane.

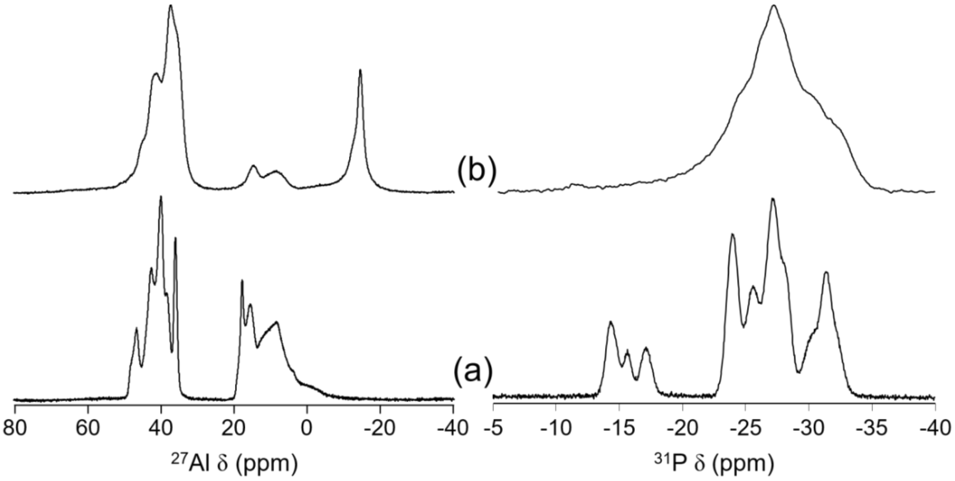

Supplementary Figure 3. 18.8 T ^{27}Al MAS (left) and ^{31}P MAS (right) NMR spectra of (a) PST-5 and (b) PST-6. In the ^{27}Al NMR spectrum of PST-5, six T_d peaks (50-35 ppm) and multiple, apparently overlapping penta-coordinated peaks (20--10 ppm) are observed. The ^{27}Al NMR spectrum of PST-6 shows multiple tetrahedral peaks (50-35 ppm), a broad penta-coordinated peak (20-5 ppm) and a sharp octahedral peak (-10--20 ppm), which indicates the structure of PST-5 and -6 are very different. However, although the second-order quadrupolar broadening of the resonances is reduced significantly, it is still not completely eliminated. The 18.8T ^{31}P MAS spectrum of PST-5 shows at least seven fully condensed $P(OAl)_4$ peaks (-20 to -35 ppm) and three partially hydrolyzed $P(H_2O)_x(OAl)_y$ peaks (-10 to -20 ppm), while the PST-6 spectrum shows four overlapping $P(OAl)_4$ peaks (-20 to -35 ppm). Unfortunately, it was not possible to correlate the local structural information obtained by the ^{31}P MAS NMR spectrum of PST-5 with mean Al-O-P bond angles of the 18 crystallographically distinct P atoms obtained from *c*RED or PXRD data. This is because PST-5 has a number of unusual penta-coordinated Al atoms which adopt trigonal bipyramidal geometry.

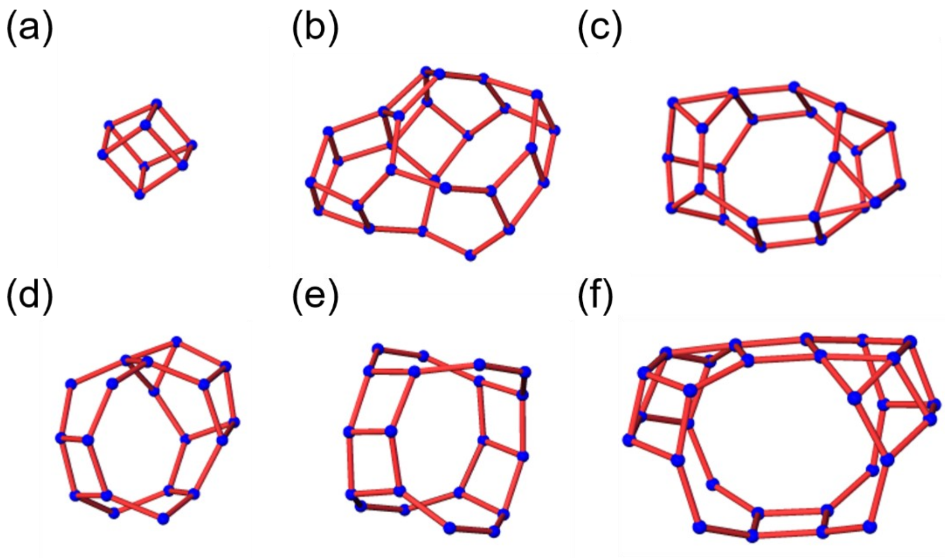

Supplementary Figure 4. Building units of PST-5. (a) [4^6], (b) [$3.4^9.5.6^2.8^3$], (c) [$3^2.4^4.5^6.8^2$], (d) [$3.4^2.5^3.6^2.8^2$], (e) [$4^4.8^2.10^2$] and (f) [$3^2.4^8.5^2.8^2.10^2$]. Oxygen atoms were removed for clarity.

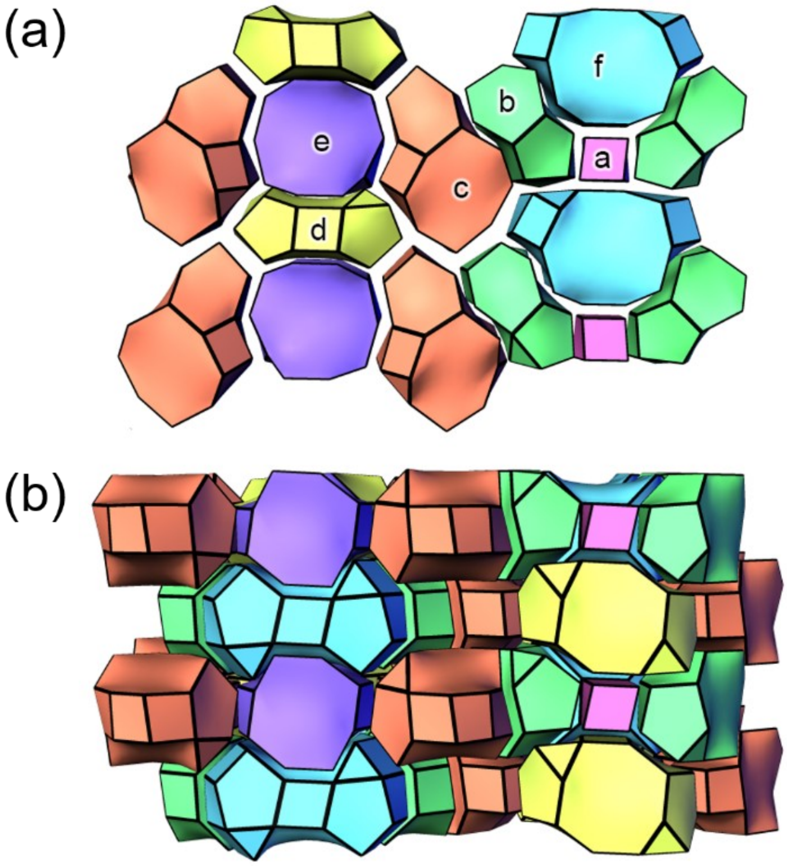

Supplementary Figure 5. Topology of PST-5. (a) The basic layer of PST-5. It is composed of the six different building units (a-f) that are shown in Figure S19. (b) The topology of the interlayer connection of two PST-5 layers.

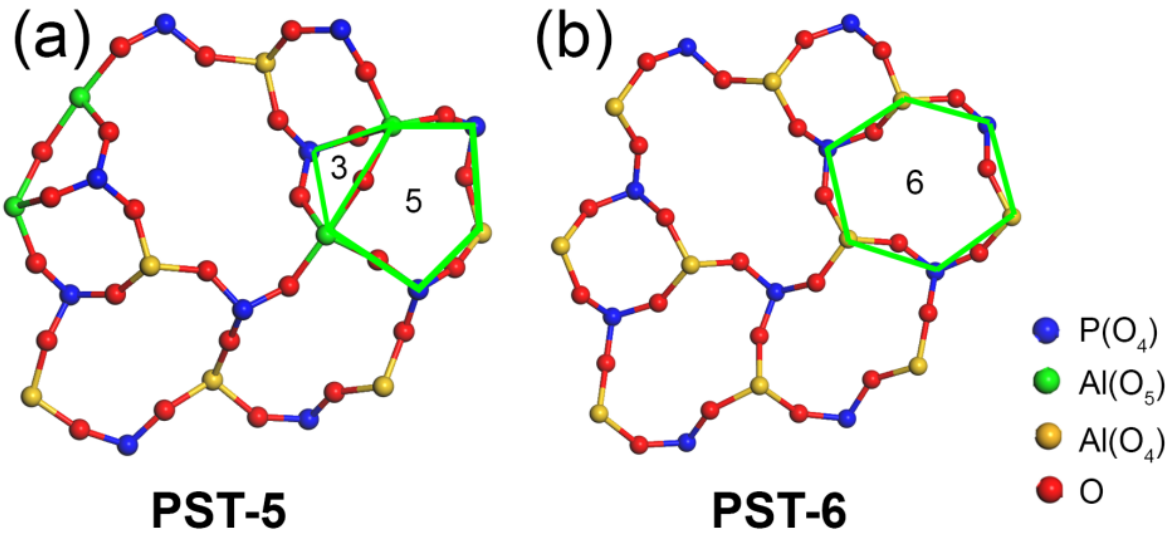

Supplementary Figure 6. (a) The structure of PST-5 with a 3- ring and a 5-ring as marked. (b) The structure of PST-6 with a 6-ring as marked. The 3- and 5-rings transform to a 6-ring after the removal of bridging OH groups.

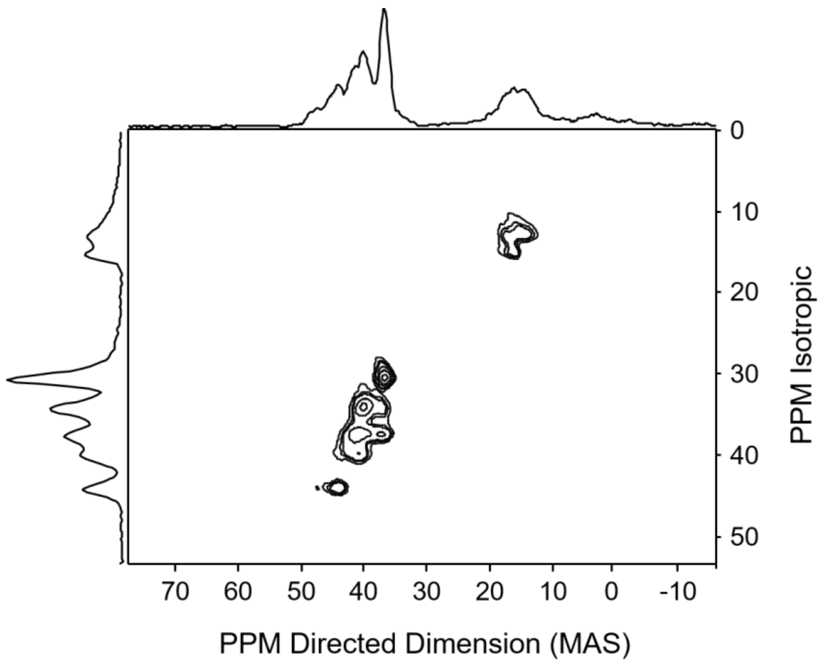

Supplementary Figure 7. 11.7T ^{27}Al 3QMAS NMR spectrum of hydrated PST-5. The ^{27}Al MAS spectrum is shown at the vertical projection and the isotropic spectrum at the horizontal projection. The vertical projection is complicated by the unaveraged quadrupolar broadening. The horizontal projection clearly shows at least five T-sites ($\delta \sim 45$ - 30 ppm region) and two penta-coordinated sites ($\delta \sim 20$ ppm) in the PST-5 framework. The breadth and symmetry of the contours in the 2D plot qualitatively indicate the size and asymmetry of the quadrupole coupling constant for each crystallographic T-site.

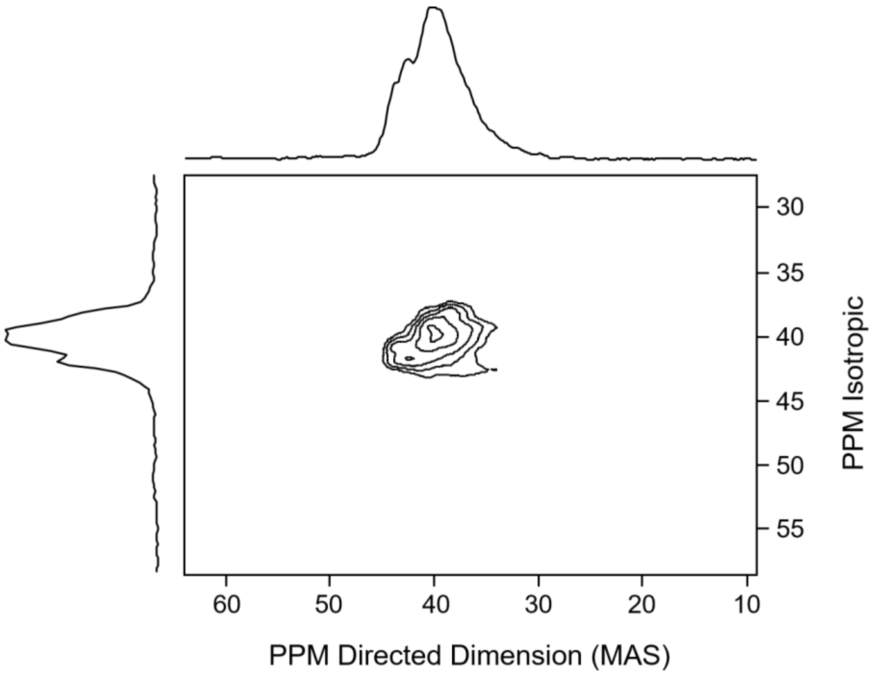

Supplementary Figure 8. 11.7T ^{27}Al 3QMAS NMR spectrum of dehydrated PST-6. The ^{27}Al 3QMAS NMR spectrum of dehydrated PST-6 is characterized by four isotropic tetrahedral ^{27}Al peaks. What appears as three T_d peaks in the MAS spectrum is actually composed of four T_d peaks of differing CQ value and very similar chemical shifts.

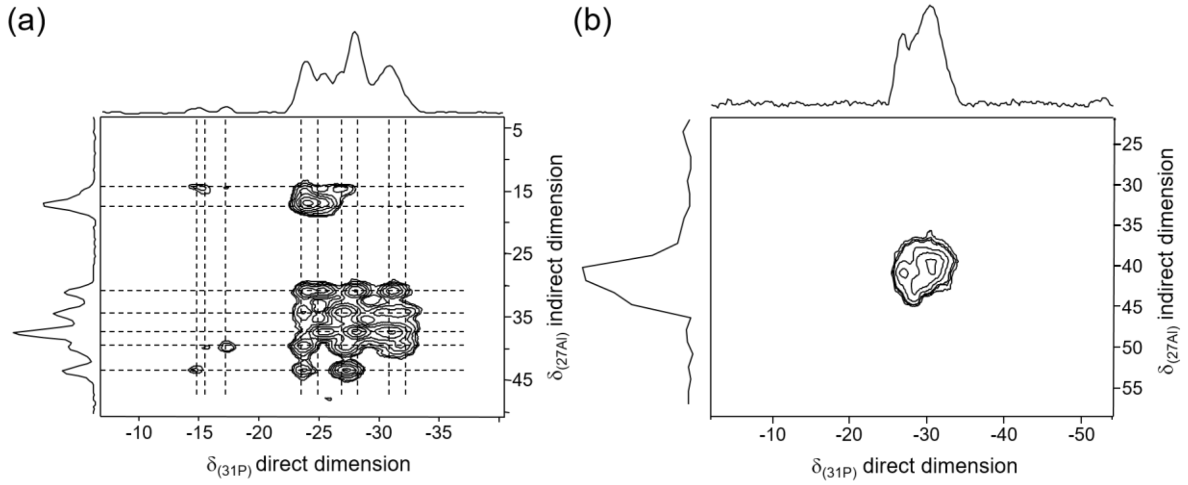

Supplementary Figure 9. 11.7 T ^{27}Al → ^{31}P 3QHETCOR MAS NMR spectra of (a) PST-5 and (b) PST-6. The 11.7 T 2D ^{27}Al → ^{31}P MQHETCOR NMR was applied to obtain more detailed information because the ^{27}Al-^{31}P dipolar couplings could add another dimension that increases the resolution and improves the spectral detail. Because this experiment is based on the transfer of magnetization from ^{27}Al to ^{31}P, the only ^{27}Al and ^{31}P nuclei that could be detected are those dipolar coupled. Thus, cross peaks in the 2D contour plot provide Al-P connectivity information. The dashed lines in (a) highlight the major couplings and permit the determination of internuclear P-Al connectivities in the three dimensional framework structure. The ^{31}P resolution is greatly enhanced in the MQHETCOR due to this dipolar editing effect. The PST-6 case is very challenging because of the very narrow chemical shift range for the multiple T_d Al and P sites in its framework. Even with this resolution limitation, the shape of the cross peak contours in (b) shows that the P peaks have slightly different Al connectivities.

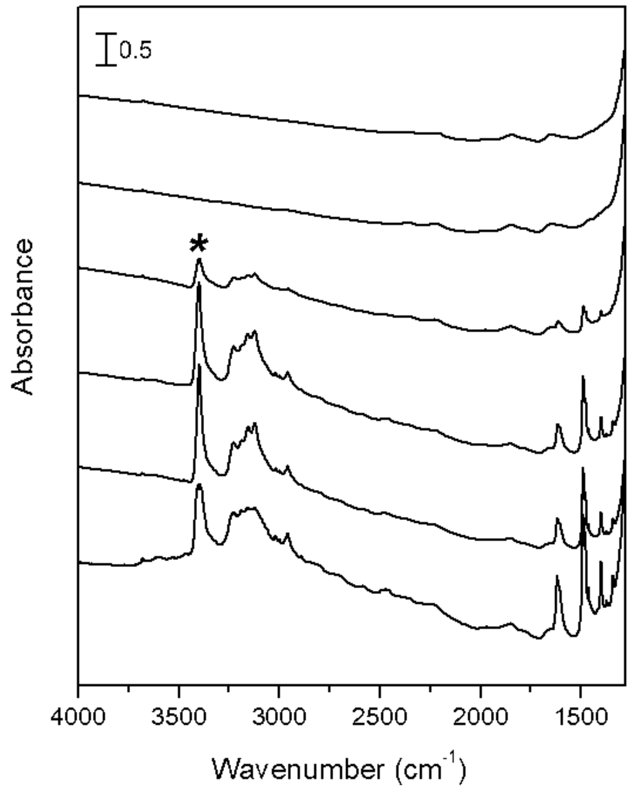

Supplementary Figure 10. IR spectra in the 1400-4000 cm^{-1} region of as-made PST-5 measured *in-situ* at different temperatures. Bottom to top: room temperature, 100, 200, 250, 300 and 400 °C. A sharp band at 3398 cm^{-1} (highlighted by asterisk) indicates bridging Al-OH-Al groups[14,15].

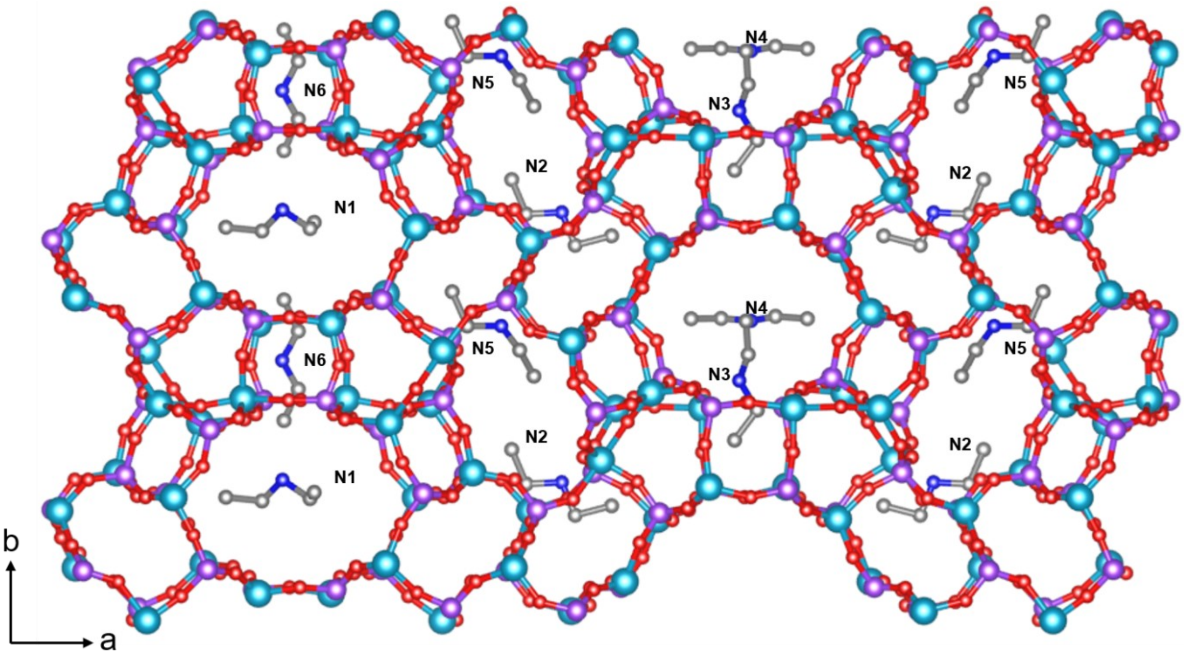

Supplementary Figure 11. The refined DEA and water molecules locations and orientations in the PST-5 framework. Al, cyan; P, violet; O, red; C, grey; N, blue.

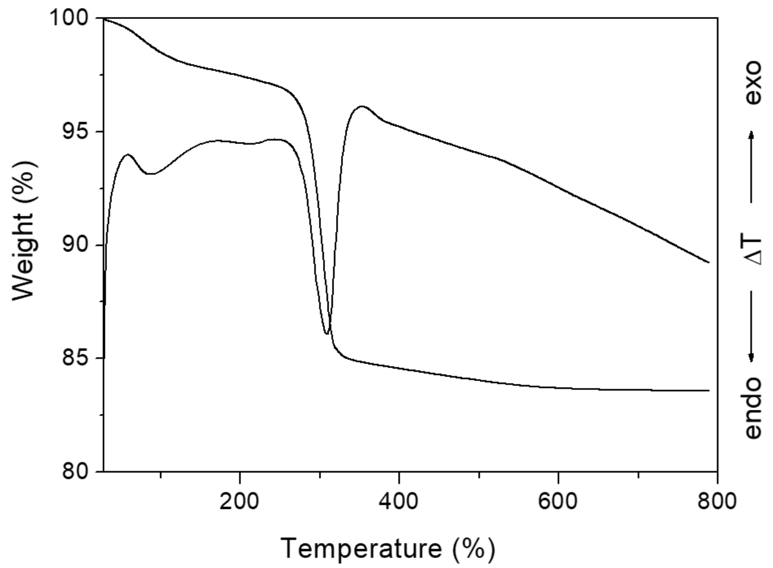

Supplementary Figure 12. TGA/DTA curves of PST-5 prepared in the presence of DEA as an OSDA. The large weight loss (~ 12 wt%) between 250 and 350 °C accompanied by a sharp endothermic peak is a result of the dehydroxylation and combustion of OSDA molecules. The lack of significant exothermic weight losses in the TGA/DTA curves can be rationalized by suggesting that the exothermic signal, which is expected from the oxidation of occluded OSDAs, was masked by the signal from the overwhelming endothermic reaction.

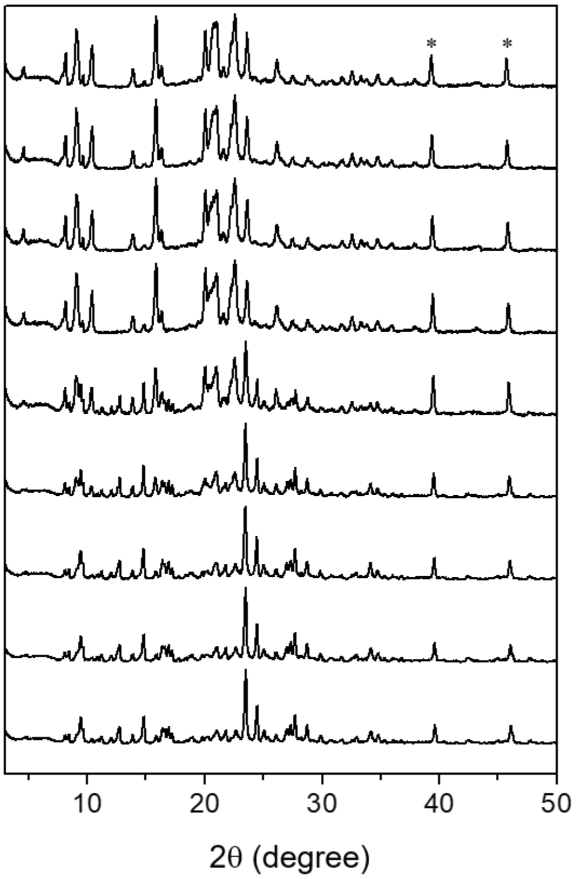

Supplementary Figure 13. Powder XRD patterns of PST-5 recorded during *in-situ* heating under vacuum to a residual pressure of 0.7 Pa at different temperatures. Bottom to top: room temperature, 100, 200, 300, 400, 500, 600, 700 and 800 °C. X-ray diffraction peaks from the Pt sample holder are marked by asterisks.

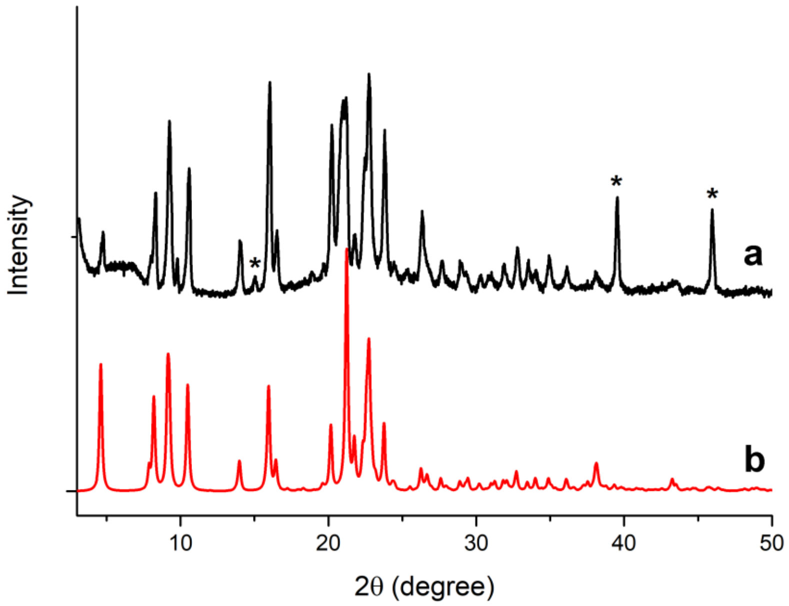

Supplementary Figure 14. (a) *In-situ* PXRD pattern of dehydrated PST-6 obtained after heating PST-5 under vacuum at 800 °C for 1 hour. (b) Simulated PXRD pattern from the PSI framework. X-ray diffraction peaks from minor impure phase and Pt sample holder are marked by asterisks.

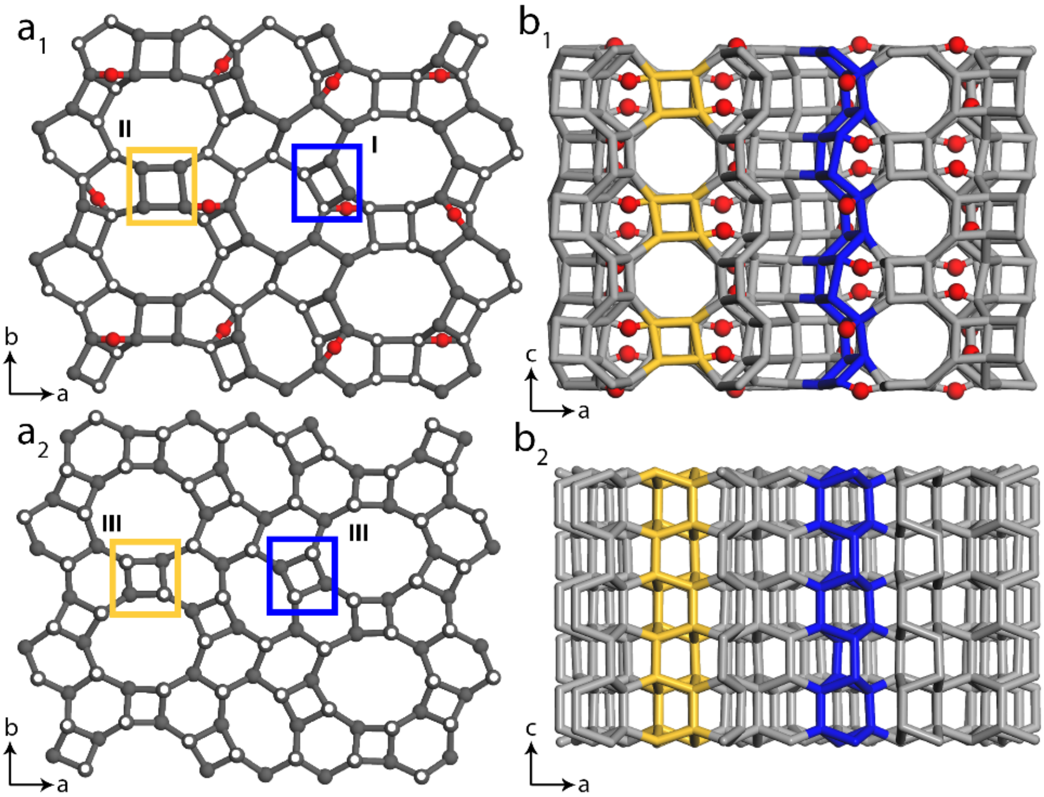

Supplementary Figure 15. (a_1 and a_2) Both structures are built from the same building layer containing 4-, 6-, 8- and 10-rings (if the -OH group is not considered). The orientations of the $(Al,P)O_4$ tetrahedra are different in the two structures. The solid and hollow circles indicate between vertices that connect upwards and downwards, respectively. The red atoms in PST-5 correspond to the OH groups bridging two Al atoms. These Al atoms are five-coordinated. (b_1) Structural models of PST-5 along [010] direction, showing double-crankshaft chains (*dcc*s; blue), double 4-rings (*d4r*s; yellow). (b_2) Structural models of PST-6 along [010] direction showing narsarsukite-type chains (*nsc*s). Red spheres, oxygen atoms.

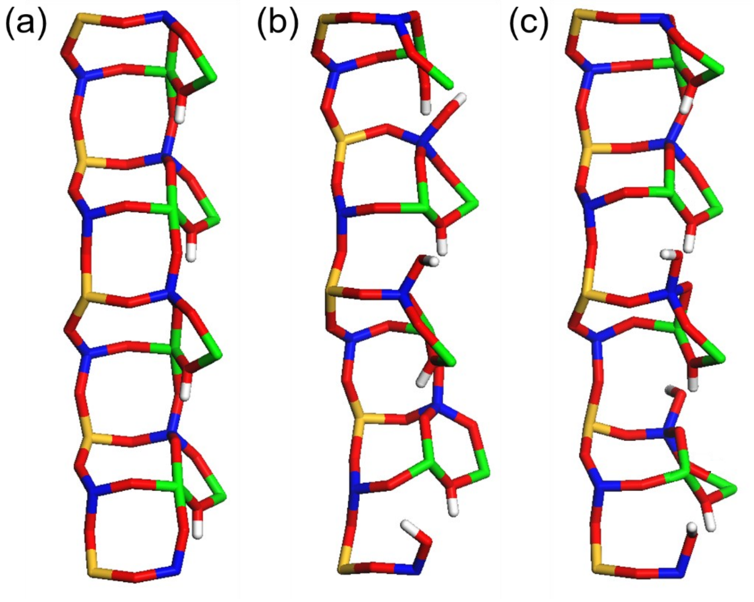

Supplementary Figure 16. Molecular dynamics simulation of the double-crankshaft unit in PST-5 containing extra-framework hydronium ions during transformation. (a) Section of double-crankshaft unit (*dcc*) from the geometry-optimized structure at the start of the simulation, which also shows adjacent 3-ring units; (b) corresponding section of *dcc* after 10 ps of simulation, showing ruptured Al-O-P linkages in the *dcc* units; (c) a different *dcc* section from the simulation. At this stage in the simulation, the 3-membered rings are still largely intact. Color code: green, initially 5-coordinated aluminum atoms; yellow, 4-coordinated aluminum atoms; blue, phosphorus atoms; red, oxygen atoms; white, hydrogen atoms.

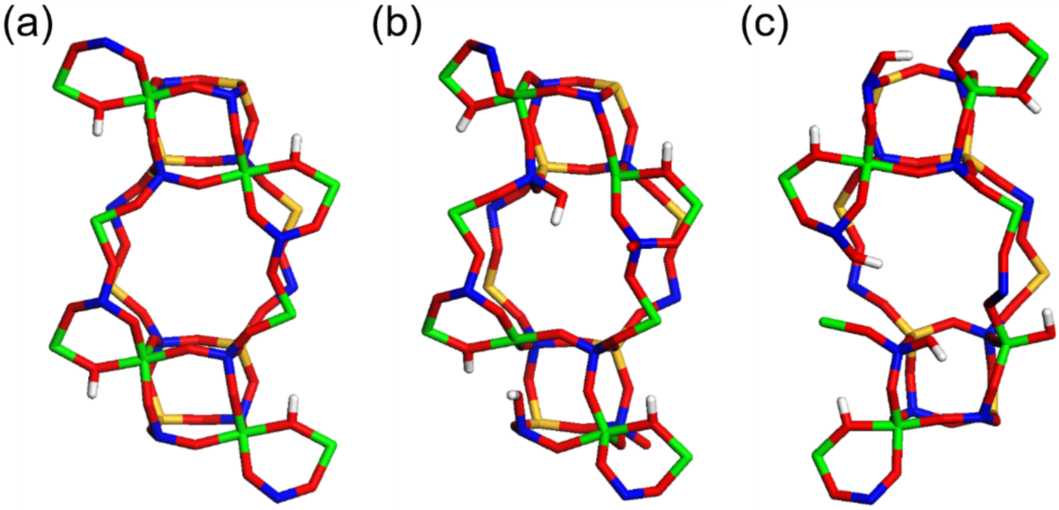

Supplementary Figure 17. Molecular dynamics simulation of the double 4-ring unit in PST-5 containing extra-framework hydronium ions during transformation. (a) Selection of the geometry-optimized structure showing two *d4r* units with connecting 6-rings at the start of the simulation, which also shows the adjacent 3-ring units; (b) corresponding sections of the structure after 10 ps of simulation, showing ruptured Al-O-P linkages in the *d4r* units and elsewhere; (c) a different section of the structure from the simulation. At this stage in the simulation, the 3-membered rings are still largely intact. Color code: green, initially 5-coordinated aluminum atoms; yellow, 4-coordinated aluminum atoms; blue, phosphorus atoms; red, oxygen atoms; white, hydrogen atoms.

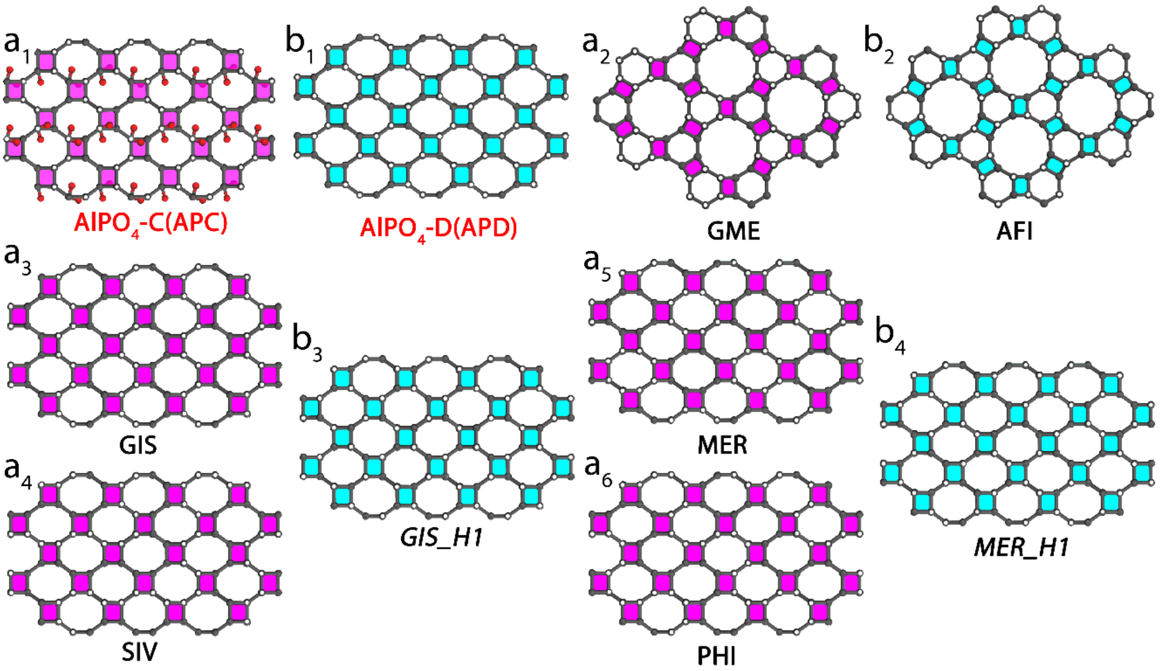

Supplementary Figure 18. (a_1-a_6) Projections of the group I *dcc*-containing frameworks (APC, GME, GIS, SIV, MER, and PHI) viewed along the chain direction. (b_1) Projection of the APD framework after topotactic transformation by changing *dcc* to *nsc*. It has the same framework projection as that of APC, but differ in the 3D connectivity along the projection. (b_2) Replacing *dcc* with *nsc* in GME and keeping the orientations result in AFI framework. (b_3) The same hypothetical structures of *GIS_H1* was generated by replacing *dcc* with *nsc* in GIS and SIV frameworks and keeping the orientations. (b_4) The same hypothetical structures of *MER_H1* was generated by replacing *dcc* with *nsc* in MER and PHI frameworks and keeping the orientations. Magenta: dcc; cyan: nsc. The solid and hollow circles indicate vertices that connect upwards and downwards, respectively.

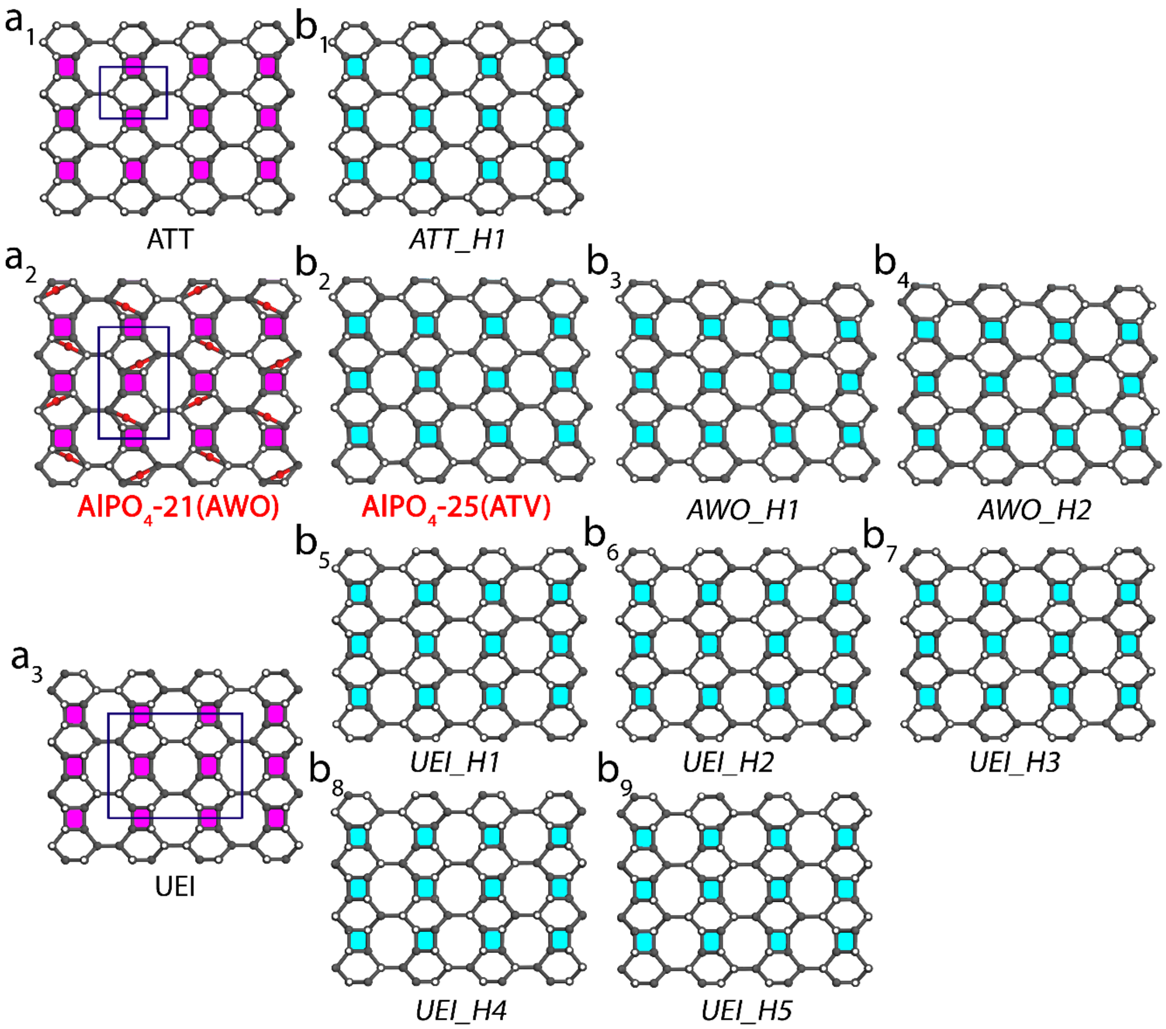

Supplementary Figure 19. (a_1-a_3) Projections of the group II *dcc*-containing frameworks (ATT, AWO and UEI) viewed along the chain direction. (b_1-b_9) Hypothetical structures of *ATT_H1*, *AWO_H1*, *UEI_H1-5* were generated by replacing *dcc* with *nsc* in ATT, AWO, and UEI frameworks, respectively and keeping the same orientations. Based on their different orientations of *nsc*, as well as the inversion of neighboring atoms, one parent structure can generate several different hypothetical structures with new topologies. The number of hypothetical structures depends on the complexity of the parent structure. Magenta: *dcc*; cyan: *nsc*. The solid and hollow circles indicate vertices that connect upwards and downwards, respectively. The marked unit cells represent those of corresponding the *nsc*-containing structures.

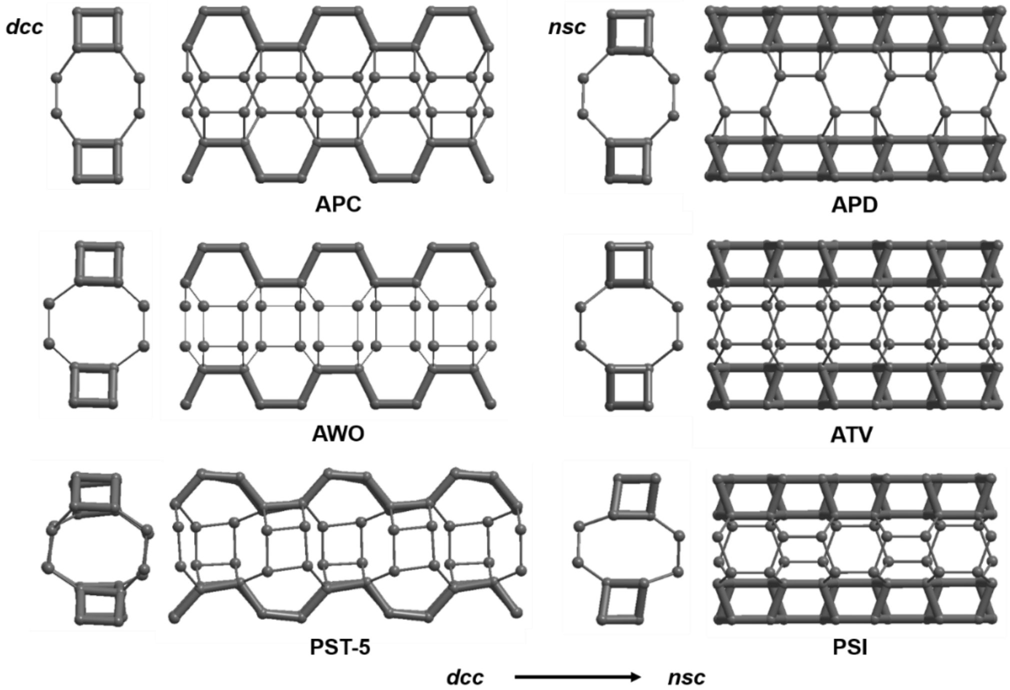

Supplementary Figure 20. The connectivity of the *dcc* and *nsc* in the frameworks viewed in two perpendicular directions. The single 4-ring columns in AWO and PST-5 are transformed to single crankshaft chains.

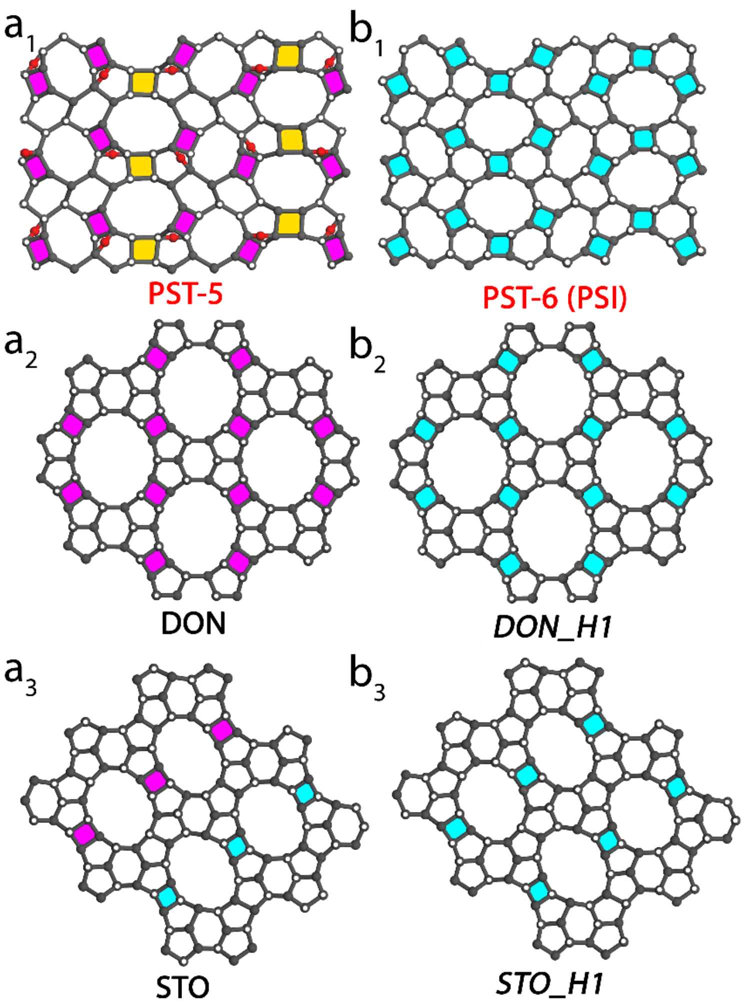

Supplementary Figure 21. (a_1-a_3) Projections of the group III *dcc*-containing frameworks (PST-5, DON, and STO). (b_1) Projections of the PSI framework after topotactic transformation by changing *dcc* to *nsc*. It has the same framework projection as PST-5, but differ in the 3D connectivity along the projection. (b_2 and b_3) Examples of hypothetical structures of *DON_H1*, and *STO_H1*, which were generated by replacing *dcc* with *nsc* in DON, and STO frameworks, respectively and keeping the same orientations. Based on the increasing structural complexity, much more possible hypothetical structures with new topologies can be generated. Magenta: *dcc*; cyan: *nsc*; yellow: *d4r*. The solid and hollow circles indicate vertices that connect upwards and downwards, respectively.

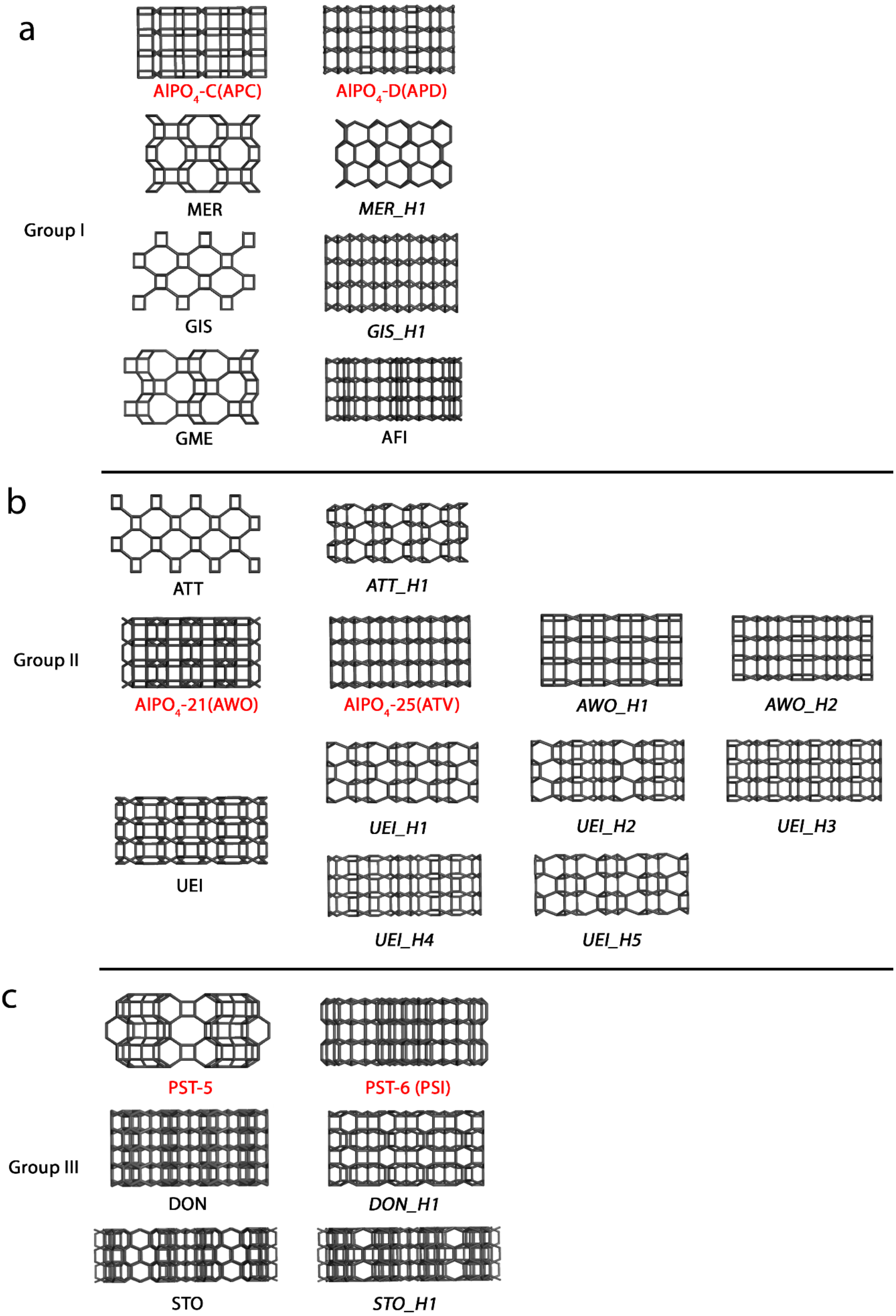

Supplementary Figure 22. Known and hypothetical zeolite structures in group I (a), group II (b), and group III (c) that can be generated by 3D-3D topotatic *dcc* to *nsc* transformations viewed perpendicular to the chains. The frameworks where the transformation has been observed are highlighted in red. In group I, *GIS-H1* and *MER-H1* can also be generated from PHI and SIV, respectively.

Supplementary Table 1. The unit cell parameters and framework densities of PST-5 and -6.

PST-	*a* (Å)	*b* (Å)	*c* (Å)	Space group	FD (T-atom/1000 $Å^3$)
PST-5	36.5952	21.8027	10.2693	*Pb*2*b*	18.2
PST-6	38.2793	22.4638	8.36197	*Pba*2	19.6

Supplementary Table 2. Experimental parameters for *c*RED data collection and crystallographic data for as-made PST-5.

Wavelength (Å)	0.0251
Total range (°)	-64.1-28.5
Rotation speed (° s^{-1})	0.45
Exposure time/frame (s)	0.4
Total number of frames	507
Data collection time (min)	3
Resolution (Å)	0.85
Crystal system	Orthorhombic
Space group	*Pb*2*b* (No. 27)
Unit cell *a*, *b*, *c* (Å)	37.917(8), 21.465(4), 10.048(2)
Volume (Å)	8178(3)
Completeness (%)	69.4
No. reflections in *c*RED	9205
No. unique reflections	5251
No. observed reflections (I > 2 sigma(I))	5009
R_1 (I > 2 sigma(I))	0.278
R_1 (all reflections)	0.318
Goof	1.46

*Hydrogen atoms were not included in the refinement.

Supplementary Table 3. PXRD data collection and crystallographic data for the Rietveld refinement of as-made PST-5.

Refined structure	$|(C_4H_{12}N^+)_{16.0}|$ $[Al_{72}P_{72}O_{288}(OH^-)_{16}]$
Symmetry	Orthorhombic
Space group	*Pb*2*b*
a (Å)	36.59450(18)
b (Å)	21.80272(9)
c (Å)	10.26930(4)
Unit cell volume ($Å^3$)	8193.66(6)
Diffractometer	Beamline 9B, PAL
Wavelength (Å)	1.54740
2θ scan range (°)	4.0-124.5
No. of contributing reflections	4322
No. of geometric restraints	438
No. of refined parameters	437
R_{wp} (%)	9.2
R_F^2 (%)	8.6
χ^2	12.8

Supplementary Table 4. Observed 3D-3D transformations of *dcc*-containing zeolites to *nsc*-containing zeolites. Unit cell parameter along the chains are highlighted in **bold**.

Chain type[a]	Framework type	*a* (Å)	*b* (Å)	*c* (Å)	*β* (°)	Space group
Group I: *dcc* only						
dcc	$AlPO_4$-C	19.821	10.028	**8.936**	90	*Pbca* (61)
nsc	AlPO-D	19.187	**8.576**	9.804	90	$Pca2_1$ *(29)*
Group II: *dcc* + pair of additional TO_4						
dcc	AlPO-21	**10.3307**	17.5241	8.6757	123.369	$P2_1/a$ (14)
nsc	AlPO-25	9.4489	15.2028	**8.4084**	90	*Acmm* (67)
Group III: *dcc*/*d*4*r* + other CBUs[b]						
dcc	PST-5	36.5956	21.8027	**10.2693**	90	*Pb*2*b* (59)
nsc	PST-6	38.2793	22.4638	**8.3620**	90	*Pba*2 (32)

[a] Framework Type Code commissioned by International Zeolite Association.
[b] Composite building units.

Supplementary Table 5. Structural information on the hypothetical zeolite structures generated by chain replacement on known zeolites. Unit cell parameter along the chains are highlighted in bold. The unit cell settings have been changed to facilitate the comparison. The space groups are also changed accordingly.

Chain type[a]	Framework type	*a* (Å)	*b* (Å)	*c* (Å)	*β* (°)	Space group
Group I: *dcc* only						
dcc	APC	19.3560	**8.9920**	10.3920	90	*Ccme* (64)]
nsc	APD	20.0600	**8.7240**	10.1660	90	*Ccme* (64)
dcc	GIS	9.8010	**9.8010**	10.1580	90	$I4_1/amd$ (141)
nsc	*GIS_H1*	9.8533	**8.3045**	9.6790	90	$Pcm2_1$ (26)
dcc	SIV	14.0754	**9.8768**	28.1314	90	*Ccmm* (63)
nsc	*GIS_H1*	9.8533	**8.3045**	9.6790	90	$Pcm2_1$ (26)
dcc	MER	14.0120	14.0120	**9.9540**	90	*I*4/*mmm* (139)
nsc	*MER_H1*	7.1001	13.9546	**8.3658**	90	*Am*2*m* (38)
dcc	PHI	14.0460	14.0640	**9.8900**	90	*Cmcm* (63)
nsc	*MER_H1*	7.1001	13.9546	**8.3658**	90	*Am*2*m* (38)
dcc	GME	13.6720	13.6720	**9.8500**	90	$P6_3/mcc$ (194)
nsc	AFI	13.8270	13.8270	**8.5800**	90	*P*6/*mcc* (192)
Group II: *dcc* + pair of additional TO_4						
dcc	ATT	7.5140	**9.9800**	9.3690	90	*Pmma* (51)
nsc	*ATT_H1*	7.5618	**8.4178**	9.3645	90.26	*Pm* (6)
dcc	AWO	15.0350	**9.1010**	19.2410	90	*Ccme* (64)
nsc	ATV	15.3110	**8.5790**	9.6610	90	*Cmme* (67)
nsc	*AWO_H1*	15.1979	**8.3297**	9.4020	90.43	*Pm* (6)
nsc	*AWO_H2*	15.4335	**8.4380**	9.3455	90	*Pmma* (51)
dcc	UEI	15.1067	**9.3507**	19.4603	90	*F*2*mm* (42)
nsc	*UEI_H1*	15.0541	**8.4189**	18.8208	90	*Pcmn* (62)
nsc	*UEI_H2*	15.1284	**8.4434**	18.7081	90.19	*Pm* (6)
nsc	*UEI_H3*	15.0489	**8.4433**	18.6432	91.29	*Am* (8)
nsc	*UEI_H4*	15.0040	**8.4433**	18.8048	89.09	*C*2/*m* (12)
nsc	*UEI_H5*	15.2032	**8.4376**	18.6949	90	*Amma* (63)
Group III: *dcc*/*d*4*r* + other CUBs[b]						
dcc	PST-5[c]	38.1844	**9.3256**	11.0935	90	*Pmmn* (59)
nsc	PSI	37.7574	**8.2625**	22.3452	90	*Aema* (64)
dcc	DON	18.8900	**8.4690**	23.3650	90	*Bmmb* (63)
nsc	*DON_H1*	18.9817	**8.4135**	23.1264	90	*Bmm*2 (38)
dcc	STO	29.8857	**8.3897**	24.7314	90	*P*2/*m* (10)
nsc	*STO_H1*	29.7581	**8.4414**	24.8963	105.37	*P*2/*m* (10)

[a] Framework Type Code commissioned by International Zeolite Association.

[b] Composite building units.

[c] Idealized framework of PST-5.

Supplementary Table 6. LID calculation results for the hypothetical zeolite structures generated by chain replacement on known zeolites.

	LID criteria[a] (Å)							
Hypothetical structure	$\varepsilon_{<OO>}$ [< 0.0009]	$\varepsilon_{<TT>}$ [< 0.0046]	σ_{TO} [< 0.0196]	σ_{OO} [< 0.0588]	σ_{TT} [< 0.0889]	R_{TO} [< 0.0634]	R_{OO} [< 0.2746]	R_{TT} [< 0.3332]
GIS_H1	0.0001	0.0019	0.0055	0.0201	0.0282	0.0184	0.0698	0.0932
MER_H1	0.0001	0.0003	0.0051	0.0197	0.0297	0.0184	0.0644	0.0978
ATT_H1	0.0001	0.0018	0.0054	0.0233	0.0253	0.0178	0.0973	0.1049
AWO_H1	0.000013	0.0013	0.0061	0.0267	0.0401	0.0323	0.1243	0.1987
AWO_H2	0.0001	0.0004	0.0051	0.0285	0.0449	0.0294	0.1049	0.1442
UEI_H1	0.0001	0.0014	0.0059	0.0240	0.0249	0.0237	0.0961	0.0838
UEI_H2	0.0000332	0.0013	0.0065	0.0254	0.0303	0.0288	0.1059	0.1091
UEI_H3	0.0001	0.0018	0.0059	0.0228	0.0284	0.0246	0.0888	0.0880
UEI_H4	0.00000491	0.0016	0.0064	0.0233	0.0258	0.0244	0.0962	0.0944
UEI_H5	0.0001	0.0010	0.0079	0.0271	0.0281	0.0242	0.0954	0.0763
STO_H1	0.000000158	0.0017	0.0056	0.0257	0.0327	0.0265	0.1316	0.1476
DON_H1	0.00000307	0.0015	0.0079	0.0275	0.0535	0.0315	0.1379	0.1984

[a] The numbers in square brackets indicate the standard values of feasible structures.[34]

Supplementary References

1. Frydman, L. & Harwood, J. S. Isotropic Spectra of Half-Integer Quadrupolar Spins from Bidimensional Magic-Angle Spinning NMR. *J. Am. Chem. Soc.* **117**, 5367–5368 (1995).
2. Medek, A., Harwood, J. S. & Frydman, L. Multiple-Quantum Magic-Angle Spinning NMR: A New Method for the Study of Quadrupolar Nuclei in Solids. *J. Am. Chem. Soc.* **117**, 12779–12787 (1995).
3. Fernandez, C., Morais, C., Rocha, J. & Pruski, M. High-Resolution Heteronuclear Correlation Spectra between 31P and 27Al in Microporous Aluminophosphates. *Solid State Nucl. Magn. Reson.* **21**, 61–70 (2002).
4. Wan, W., Sun, J., Su, J., Hovmöller, S. & Zou, X. Three-dimensional rotation electron diffraction: software RED for automated data collection and data processing. *J. Appl. Crystallogr.* **46**, 1863–1873 (2013).
5. Zhang, D., Oleynikov, P., Hovmöller, S. & Zou, X. Collecting 3D electron diffraction data by the rotation method : Zeitschrift für Kristallographie International journal for structural, physical, and chemical aspects of crystalline materials. *Z Krist.* **225**, 94–102 (2010).
6. Simancas, J. *et al.* Ultrafast Electron Diffraction Tomography for Structure Determination of the New Zeolite ITQ-58. *J. Am. Chem. Soc.* **138**, 10116–10119 (2016).
7. Kabsch, W. Integration, scaling, space-group assignment and post-refinement. *Acta Crystallogr. D Biol. Crystallogr.* **66**, 133–144 (2010).
8. Sheldrick, G. M. A short history of SHELX. *Acta Crystallogr. A* **64**, 112–122 (2008).
9. Rietveld, H. M. A profile refinement method for nuclear and magnetic structures. *J. Appl. Crystallogr.* **2**, 65–71 (1969).
10. Larson, A. C. & von Dreele, R. B. General Structure Analysis System GSAS, Los Alamos National Laboratory, Los Alamos, NM, 2000. (2000).
11. Toby, B. H. EXPGUI, a graphical user interface for GSAS. *J. Appl. Crystallogr.* **34**, 210–213 (2001).
12. Hastings, J. B., Thomlinson, W. & Cox, D. E. Synchrotron X-ray powder diffraction. *J. Appl. Crystallogr.* **17**, 85–95 (1984).
13. Favre-Nicolin, V. & Černý, R. FOX, `free objects for crystallography': a modular approach to ab initio structure determination from powder diffraction. *J. Appl. Crystallogr.* **35**, 734–743 (2002).
14. Namkung, J. S., Hoke, M., Rogowski, R. S. & Albin, S. FT-IR Optical Fiber Remote Detection of Aluminum Hydroxide by Evanescent Wave Absorption Spectroscopy. *Appl. Spectrosc.* **49**, 1305–1310 (1995).
15. Cheng, S., Tzeng, J.-N. & Hsu, B.-Y. Synthesis and Characterization of A Novel Layered Aluminophosphate of Kanemite-like Structure. *Chem. Mater.* **9**, 1788–1796 (1997).
16. Park, G. T., Jo, D., Ahn, N. H., Cho, J. & Hong, S. B. Synthesis and Structural Characterization of a CHA-type AlPO4 Molecular Sieve with Penta-Coordinated Framework Aluminum Atoms. *Inorg. Chem.* **56**, 8504–8512 (2017).
17. Chippindale, A. M. *et al.* A three-dimensional framework aluminophosphate (CH3NH3)+[Al3P3O13H]−. *Acta Crystallogr. C* **50**, 1537–1540 (1994).

18. Bennett, J. M., Cohen, J. M., Artioli, G., Pluth, J. J. & Smith, J. V. Crystal structure of AlPO4-21, a framework aluminophosphate containing tetrahedral phosphorus and both tetrahedral and trigonal-bipyramidal aluminum in 3-, 4-, 5-, and 8-rings. *Inorg. Chem.* **24**, 188–193 (1985).
19. Lee, J. K. *et al.* An Aluminophosphate Molecular Sieve with 36 Crystallographically Distinct Tetrahedral Sites. *Angew. Chem. Int. Ed.* **53**, 7480–7483 (2014).
20. Guo, P., Wan, W., McCusker, L. B., Baerlocher, C. & Zou, X. On the relationship between unit cells and channel systems in high silica zeolites with the "butterfly" projection : Zeitschrift für Kristallographie - Crystalline Materials. *Z Krist.* **230**, 301–309 (2015).
21. about [CP2K Open Source Molecular Dynamics]. https://www.cp2k.org/.
22. VandeVondele, J. *et al.* Quickstep: Fast and accurate density functional calculations using a mixed Gaussian and plane waves approach. *Comput. Phys. Commun.* **167**, 103–128 (2005).
23. VandeVondele, J. & Hutter, J. Gaussian basis sets for accurate calculations on molecular systems in gas and condensed phases. *J. Chem. Phys.* **127**, 114105 (2007).
24. LIPPERT, B. G., HUTTER, J. & PARRINELLO, M. A hybrid Gaussian and plane wave density functional scheme. *Mol. Phys.* **92**, 477–488 (1997).
25. Perdew, J. P., Burke, K. & Ernzerhof, M. Generalized Gradient Approximation Made Simple. *Phys. Rev. Lett.* **77**, 3865–3868 (1996).
26. Baerlocher, C., Hepp, A. & Meier, W. M. *DLS-76: A Program for the Simulation of Crystal Structures by Geometric Refinement*. (Institute of crystallography and petrography - ETH, 1978).
27. Goedecker, S., Teter, M. & Hutter, J. Separable dual-space Gaussian pseudopotentials. *Phys. Rev. B* **54**, 1703–1710 (1996).
28. Mayo, S. L., Olafson, B. D. & Goddard, W. A. DREIDING: a generic force field for molecular simulations. *J. Phys. Chem.* **94**, 8897–8909 (1990).
29. Schröder, K.-P. *et al.* Bridging hydrodyl groups in zeolitic catalysts: a computer simulation of their structure, vibrational properties and acidity in protonated faujasites (H□Y zeolites). *Chem. Phys. Lett.* **188**, 320–325 (1992).
30. Blatov, V. A., Shevchenko, A. P. & Proserpio, D. M. Applied Topological Analysis of Crystal Structures with the Program Package ToposPro. *Cryst. Growth Des.* **14**, 3576–3586 (2014).
31. Gale, J. D. & Rohl, A. L. The General Utility Lattice Program (GULP). *Mol. Simul.* **29**, 291–341 (2003).
32. Momma, K. & Izumi, F. VESTA 3 for three-dimensional visualization of crystal, volumetric and morphology data. *J. Appl. Crystallogr.* **44**, 1272–1276 (2011).
33. Foster, M. D. *et al.* Chemically feasible hypothetical crystalline networks. *Nat. Mater.* **3**, 234–238 (2004).
34. Li, Y., Yu, J. & Xu, R. Criteria for Zeolite Frameworks Realizable for Target Synthesis. *Angew. Chem. Int. Ed.* **52**, 1673–1677 (2013).
