# Supplementary material for: 3D-3D topotactic transformation in aluminophosphate molecular sieves and its implication in new zeolite structure generation
Source: Nat Commun. 2020 Jul 28;11:3762. doi: 10.1038/s41467-020-17586-7 (PMC7387333; doi:10.1038/s41467-020-17586-7)
Supplement: Supplementary file 2 — Peer Review File [file 41467_2020_17586_MOESM2_ESM.docx]

Peer Review Information

**Journal:** Nature Communications

**Manuscript title:** Click here to enter text.
3D-3D Topotactic Transformation in Aluminophosphate Molecular Sieves and Its Implication in New Zeolite Structure Generation

**Corresponding author name(s):** Prof Ziaodong Zou

**Editorial notes:**

**Transferred manuscripts**

This manuscript has been previously reviewed at another journal that is not operating a transparent peer review scheme. This document only contains reviewer comments and rebuttal letters for versions considered at *Nature Communications*.

**Reviewer comments & decisions:**

**Reviewer comments, first version:**

Reviewers' comments:

Reviewer #1 (Remarks to the Author):

This manuscript describes the crystal structure solution of a microporous aluminophosphate, PST-5, using a fast 3-D electron diffraction technique. As PST-5 is the synthetic precursor of a related structure PST-6, this work allows understanding the transformation of PST-5 to PST-6. This understanding in combination with previous observations in the AlPO4 zeolites inspires the authors to claim that the dcc-nsc (or d4r-nsc) transformation can be exploited as a general top-down approach to produce new zeolite frameworks, and a number of hypothetical zeolite structures have been proposed accordingly.
Technically, the structural solution of PST-5 is convincing and the manuscript is well organized. My specific comments are listed below.
1. It is unclear how the bridging group is assigned to be hydroxyl (OH) instead of H2O. If it is hydroxyl, what is the charge balancing species to compensate the negative charge? This point should be elaborated.
2. The authors mentioned that “its framework was solved ab initio by direct methods and followed by refinement against cRED data”. In the later section, it is mentioned that the OSDA positions were determined by refinement against synchrotron PXRD data. If synchrotron PXRD data is available, why did the authors refine the structure using cRED data? What are the main difference between the refinement results based on X-ray and electron diffraction? This should be discussed and clarified in the main text if the authors choose to use both data in the paper.
3. The difficulty of resolving the structure of PST-5 by 3-D electron diffraction (ED) lies in its sensitivity to the electron beam irradiation, and this problem has been overcome in this study by using fast 3-D ED. It would be better if the authors can estimate the total electron doses that PST-5 can withstand before its structure is damaged.
4. The authors attribute the structure transformation to the weak bond associated with penta-coordinated AlO4-OH-AlO4 pairs. This hypothesis has been verified by the MD simulation. In fact, all successful dcc-nsc structural transformations (PST-5/PST-6; AWO/ATV; APC/APD) involve penta- or hexa-coordinated Al with bridging/terminal hydroxyl or water groups. This fact also implies that the presence of penta- or hexa-coordinated Al is a prerequisite for the structural transformation driven by dihydroxylation. However, this prerequisite has been ignored by the authors in the later section of the manuscript, when proposing hypothetical zeolite structures, where their statements sound like the dcc-nsc structural transformation can generally take place regardless the initial state of TO4. This may not be correct, as evidenced by the lack of successful examples, despite many hypothetical structures (e.g., DON and *STO) proposed in this paper.
5. If this synthetic strategy (3D-3D topotactic transformation) is not as generally applicable as the authors claim (see the point above), I have reservations about recommending this work to be published in Nature Comm. Or at least, the authors should comment on the likelihood of dcc-nsc (or d4r-nsc) transformation in the cases that there are no penta-coordinated Al pairs in the initial structure.


Reviewer #2 (Remarks to the Author):

This is a very nice study of the transformation of a structurally hydrated AlPO4 phase upon calcination and certainly of high interest for researchers interested in formation and structure of porous materials.
The solved structure is quite convincing and consistent with observation.
However, it is not conclusively demonstrated that the resulting phase (PST-6) fully corresponds to the already published PSI framework: a thorough structure analysis of the XRD-pattern obtained after calcination could not be found. Furthermore, it is difficult to assess if the resulting phase is a fully ordered material as the X-ray data are presented in low graphical resolution and shows – as far as can be discerned – linebroadening. Linebroadening also is obvious from the NMR data, where the pattern of the calcined phase appears as a broadened, but very similar pattern compared to PST-5. To exclude the formation of a disordered framework, ED would be necessary, to clearly demonstrate no steaking occurs along any axis. Interestingly, the previously published work on PST-6 seems to indicate this material indeed is displaying stacking faults. The published ED data had shown streaking, even though this was not addressed by the authors previously.
Nonetheless, the work has merit, because this is a further and quite convincing example on the transformation of a porous structure containing 5-fold coordinated Al sites upon deep thermal dehydration. On the other hand, this observation is not novel and commonly observed in AlPO4 phases. The simultaneous occurrence of local inversion and resulting restructuring likewise is no real surprise.
The purely theoretical enumeration of hypothetical frameworks which might result from a systematic phase transformation based on the deduced layers is quite nice and appealing in a topological view, but unless a clear strategy is suggested on how a deep dehydration can be directed to one specific and pure framework it remains a very intriguing brain-game only.
In summary: This contribution has many strong points, is pleasant to read but has a number of shortcomings, which leads this reviewer to advise its rejection for Nature Communications. However, for a specialized readership the article’s content certainly is of high interest.


Reviewer #3 (Remarks to the Author):

This is a really interesting article. The ADOR method was the first to show a completely new solid-solid transition mechanism for zeolite synthesis. This paper presents a second. I have only a few comments
1) These two sentences do not appear to be logical: "In AlPO4 frameworks, the AlO4 and PO4 tetrahedra always alternate according to Löwenstein's rule32. Nevertheless, a pair of AlO4tetrahedra can be bridged by a hydroxyl group to adopt a trigonal bipyramidal-coordination, as found in AlPO4-21 (AWO)33 and AlPO4-C (APC)3" The authors need to say something like "when pentavalent Al is allowed" after "Nevertheless" to make these logical.
2) There is some confusion the text whether the pentavalent Al in PST-5 imply that dcc or d4r chains necessarily contain pentavalent Al. This should be clarified.
3) This sentence "It should be noted that molecular dynamics simulation of a hypothetical “pure” AlPO4 PST-5 structure without penta-coordinated Al atoms and bridging OH groups showed no cleavage of any framework bonds over the same timescale at 500 oC" is confusing, because the text had implied PST-5 contains pentavalent Al. What the "pure" structure is should be clarified somewhere, may in the SI.
4) Related to #2 and "This implies that new zeolite frameworks can be synthesized via exploitation of the dcc-nsctransformation" the issue of pentavalency needs to be clarified. Zeolites do not contain pentavalent T atoms.
5) It would be helpful in Figure 4,5 to have arrows pointing from parent to hypothetical transformed zeolites (as there are in Figure 3).
6) I do not seem to understand Table 1. What does FTC stand for? Which is parent and which is transformed zeolite? Also, how is the energy calculated (SLC or ab initio)?
7) As can be seen from my comments, I am unclear about the transformation mechanism for known zeolites. Are the authors saying known zeolite 1 can be come pentavalent and then can transform back to four-valent known zeolite 2? So, the intermediate state only is pentavalent? And this would occur only with Al-containing zeolites? This should all be explicitly stated.

**Author rebuttal, first version:**

This manuscript describes the crystal structure solution of a microporous aluminophosphate, PST-5, using a fast 3-D electron diffraction technique. As PST-5 is the synthetic precursor of a related structure PST-6, this work allows understanding the transformation of PST-5 to PST-6. This understanding in combination with previous observations in the AlPO4 zeolites inspires the authors to claim that the dcc-nsc (or d4r-nsc) transformation can be exploited as a general top-down approach to produce new zeolite frameworks, and a number of hypothetical zeolite structures have been proposed accordingly.

**Reviewer #1:**

Technically, the structural solution of PST-5 is convincing and the manuscript is well organized. My specific comments are listed below.

***Response***: We thank the reviewer’s appreciation of our work and the valuable comments and suggestions. We have now revised the manuscript accordingly to address all the issues raised by the reviewer. The point-to-point responses are given below.

1. It is unclear how the bridging group is assigned to be hydroxyl (OH) instead of H2O. If it is hydroxyl, what is the charge balancing species to compensate the negative charge? This point should be elaborated.

***Response***: The hydroxyl OH group was assigned based on the IR spectrum of PST-5 (Figure R1). The sharp and intense band at 3398 cm^-1^ can only be assigned to AlO-H stretching absorption from the bridging Al-OH-Al groups, not to H2O molecules, see *Appl. Spectrosc., 49, 1305 (1995); Chem. Mater., 9, 1788 (1997)*. The following text on page 3,

“PST-5 has one of the most complex zeolite structures with 18 Al, 18 P, 72 O atoms and 4 hydroxyl (OH) groups in the asymmetric unit.”

has been modified to

“PST-5 is one of the most complex zeolite structures, with 18 Al, 18 P, and 76 O atoms in the asymmetric unit. Four of the O atoms belong to hydroxyl groups and act as bridging atoms to form Al-OH-Al linkages.”

The following text on page 3

“The IR and solid-state NMR results confirmed the presence of bridging hydroxyl groups (Figs. S3-S7)”

has been moved to page 4 and modified to

1

“Solid-state NMR spectroscopy confirmed the presence of Al-O-Al linkages (Figs. S3-S6), and a sharp IR band appearing at 3398 cm^-1^ indicates the presence of bridging OH groups (Fig. S7).”

In addition, we marked the sharp IR band at 3398 cm^-1^ by an asterisk in Fig. S7 (Fig. S3 in previous version) and added “A sharp band at 3398 cm^-1^ (highlighted by asterisk) indicates bridging Al-OH-Al groups.” in the caption.

**Figure S7.** IR spectra in the 1400-4000 cm^-1^ region of as-made PST-5 measured *in-situ* at different temperatures. Bottom to top: room temperature, 100, 200, 250, 300 and 400 °C. A sharp band at 3398 cm^-1^ (highlighted by asterisk) indicates bridging Al-OH-Al groups^14,15^.

The charge balancing species are protonated diethylamine DEA•H^+^, (C2H5)2NH2+. This is supported by the Raman spectrum of PST-5 where no band corresponding to the N-H stretching mode of neutral DEA around 3320 cm^-1^ is observed (Figure R1). We further located two more DEA molecules from difference electron potential map based on the cRED data, and performed a new Rietveld refinement against the synchrotron PXRD data. The final unit cell composition of PST-5 determined from the Rietveld refinement is |(C2H5)2NH2^+^)16|[Al72P72O288(OH^-^)16], where the 16 DEA•H^+^ cations compensate for the negative charge of the bridging Al-OH-Al group.

The following text has been added on page 4.

“Raman spectrum shows that DEA molecules are protonated during PST-5 crystallization^35^. Therefore, the negative framework charges created by the bridging Al-OH-Al groups are balanced by the protonated form of the occluded OSDA molecules (DEA•H^+^), as reflected by quite similar numbers of OSDA molecules and bridging O atoms.”

2

**Figure R1*.*** Raman spectra in the 200-3500 cm^-1^ region of a) DEA, b) protonated DEA (DEA∙H^+^), c) AlPO4-21, and d) PST-5. The N-H stretching mode of neutral DEA is marked with an arrow, and the structural Raman bands of each molecular sieve are indicated by asterisks. From *Angew. Chem. Int. Ed. 53, 7480 (2014).*

2. The authors mentioned that “its framework was solved ab initio by direct methods and followed by refinement against cRED data”. In the later section, it is mentioned that the OSDA positions were determined by refinement against synchrotron PXRD data. If synchrotron PXRD data is available, why did the authors refine the structure using cRED data? What are the main difference between the refinement results based on X-ray and electron diffraction? This should be discussed and clarified in the main text if the authors choose to use both data in the paper.

***Response***: We conducted the structure refinement using cRED data to further confirm the framework structure and obtain more accurate positions and atomic displace parameters (ADPs) of individual atoms. Electron diffraction does not have peak overlapping problem and therefore provides much more independent data than PXRD. This makes it possible to refine not only the atomic positions, but also the ADPs of individual atoms, while ADPs are usually refined in groups in the Rietveld refinement against PXRD data. On the other hand, intensities from PXRD do not suffer from multiple scattering and are more reliable than those from cRED. Although we were able to determine the locations of OSDAs from the difference electron potential map using cRED data, the refinement was not stable. With PXRD data, it is possible to refine OSDA positions. Therefore, we use both X-ray and electron diffraction data to obtain as much detailed structural information as possible. We made a new Rietveld refinement of PST-5 against PXRD data, where two additional OSDAs are located from the difference electron density map to add up to totally 16 OSDAs per unit cell.

The following text on page 3,

“Its framework structure was solved *ab initio* by direct methods and followed by refinement against the *c*RED data (Table S2)”, has been modified to

3

“Its framework structure was first solved *ab initio* by direct methods. To confirm the model and obtain more accurate positions and atomic displace parameters (ADPs) of individual atoms, the model was further refined against the *c*RED data (Table S2).”

The following text on page 4, “The OSDA positions in the channels were located in both ED and PXRD and refined by Rietveld refinement against synchrotron powder XRD data (Figs. 1b and S9, Table S3, see Supplementary Information for more details)” has been modified to

“The structure model obtained by Rietveld refinement against synchrotron powder XRD data agrees well with that obtained from *c*RED data, which further confirms the framework structure of PST-5. In addition, the OSDA positions in the channels could be located from both *c*RED and PXRD data, and subsequently refined by Rietveld refinement. The final refined unit cell composition is |(C2H5)2NH2^+^)16|[Al72P72O288(OH^-^)16] (Figs. 1b and S9, Table S3, see Supplementary Information for more details).”

The manuscript and Table S3 have been updated based on the new refinement results, and the following changes have been made to the text

on page 5, “... where Al-O bond lengths (on average 1.84(9) Å) ...” to “... where Al-O bond lengths (on average 1.83(9) Å) ...”.

on page S4, Supplementary Information, “The final *R*wp and *R*p values converged at 0.093 and 0.068, respectively.” to “The final *R*wp and *R*p values converged at 0.092 and 0.067, respectively.” and “The refined unit cell composition |(C4H11N)14.0(H2O)2.0|[Al72P72O288(OH)16] ...” to “The refined unit cell composition |(C2H5)2NH2^+^)16|[Al72P72O288(OH^-^)16] ...”

We have updated the crystallographic file (cif) of PST-5 refined against PXRD data in the CCDC database with deposition number 1948775. The cif file for cRED data has also been updated.

3. The difficulty of resolving the structure of PST-5 by 3-D electron diffraction (ED) lies in its sensitivity to the electron beam irradiation, and this problem has been overcome in this study by using fast 3-D ED. It would be better if the authors can estimate the total electron doses that PST-5 can withstand before its structure is damaged.

***Response***: The total electron dose that PST-5 can withstand is estimated to be 15 e Å^-2^. The following text on page 3,

“A high-resolution *c*RED dataset of PST-5 (with resolution better than 0.85 Å) was collected from a sub-micrometer sized crystal using low dose and fast data collection (totally in 3 min) ...”, has been revised to

“A high-resolution *c*RED dataset of PST-5 (with a resolution better than 0.85 Å) was collected from a sub-micrometer sized crystal using low dose and fast data collection (in total 15 e Å^-2^ and 3 min) ...”.

4. The authors attribute the structure transformation to the weak bond associated with penta-coordinated AlO4-OH-AlO4 pairs. This hypothesis has been verified by the MD simulation. In fact, all successful dcc-nsc structural transformations (PST-5/PST-6; AWO/ATV; APC/APD) involve penta- or hexa-coordinated Al with bridging/terminal hydroxyl or water groups. This fact also implies that the presence of penta- or hexa-coordinated Al is a prerequisite for the structural transformation driven by dihydroxylation. However, this prerequisite has been ignored by the authors in the later section of the manuscript, when proposing hypothetical zeolite structures,

4

where their statements sound like the dcc-nsc structural transformation can generally take place regardless the initial state of TO4. This may not be correct, as evidenced by the lack of successful examples, despite many hypothetical structures (e.g., DON and *STO) proposed in this paper.

***Response***: The reviewer is right that the presence of penta- or hexa-coordinated Al atoms is crucial for the structural transformation. We have already pointed out this in several places in the manuscript, for example on page 2 “The transformation was initiated via the weak Al-O bonding in penta-coordinated AlO4(OH) pairs.” and on page 5 “The key to the chain transformation is the penta-coordinated Al atoms, where Al-O bond lengths (on average 1.83(9) Å) are longer than those of tetra-coordinated Al atoms (on average 1.72(1) Å)”. We agree with the reviewer that we should have made it clearer when proposing hypothetical zeolite structures.

To further clarify this point, we have made the following revisions and changed:

“The transformation was initiated via the weak Al-O bonding in penta-coordinated AlO4(OH) pairs.” on page 3 to “The transformation was initiated via the weak Al-O bonding in penta-coordinated AlO4(OH) pairs or hexa-coordinated AlO4(OH)2 units.”

“It should be noted that molecular dynamics simulation of a hypothetical “pure” AlPO4 PST-5 structure without penta-coordinated Al atoms and bridging OH groups showed no cleavage of any framework bonds over the same timescale at 500 ^o^C. “ on page 6 to

“It should be noted that the molecular dynamics simulations of an idealised, fully four-connected, AlPO4 PST-5 framework, without penta-coordinated Al atoms and bridging OH groups, showed no cleavage of any framework bonds over the same timescale at 500 ^o^C.” (page 7)

5. If this synthetic strategy (3D-3D topotactic transformation) is not as generally applicable as the authors claim (see the point above), I have reservations about recommending this work to be published in Nature Comm. Or at least, the authors should comment on the likelihood of dcc-nsc (or d4r-nsc) transformation in the cases that there are no penta-coordinated Al pairs in the initial structure.

***Response***: We believe the topotatic transformation is generally applicable for zeolite frameworks containing *dcc* with penta- and hexa-coordinated Al atoms. This has been demonstrated in three examples, PST-5/PST-6, AWO/ATV, APC/APD, despite their different levels of structural complexity. In all these cases, penta- or hexa-coordinated Al atoms are presented in *dcc* chains, which have been transformed to *nsc* chains. We also pointed out that our new strategy for rationally generating new zeolite structures is to introduce the weak-bonding, such as the penta-coordinated Al atoms in the framework.

Penta-coordinated Al atoms may be introduced into AlPO4 frameworks by selecting OSDAs. We find there is a number of synthesis conditions that penta-coordinated Al atoms have been incorporated (*Nature*, 331, 698 (1988); *Angew. Chem. Int. Ed.*, 57, 3727 (2018); Chem. Mater*.,*

12, 2517 (2000); *Stud. Surf. Sci. Catal*., 24, 271 (1985); *Micropor. Mesopor. Mat*., 57, 211 (2003); *J. Chem. Soc., Faraday Trans*., 94, 723 (1998); etc.). Moreover, we have successfully synthesized three different AlPO4 materials have penta-coordinated Al atoms, i.e. AlPO4-21, PST-5 and PST-
13, by using diethylamine as OSDA, two of them have the *dcc* chains that can be transformed. From these results, OSDA is one important factor to introduce penta-coordinated Al atoms into pure frameworks. Therefore, we draw a feasible synthesis strategy that includes the use of diamines with head groups similar to diethylamine but different chain lengths as shown in Figure

5

R2. Additionally, through charge balancing, a further protonation of the OSDAs, such as amines to aminium ions during the crystallization process could promote the formation of bridging Al-OH-Al bonds.

**Figure R2.** Suggested diamines for the synthesis of AlPO4 molecular sieves containing bridging hydroxyl groups.

Moreover, main block elements or transition metal ions, which have weaker bonding energies with O than tetra-coordinated atoms, may also be potential candidates, if the metals have ordered arrangement in zeolites. For example, the gallophosphate Mu-18 (UEI) already contains both *dcc* and penta-coordinated atoms (*Chem. Mater*, 13, 1305 (2001)).

As we discussed above, we believe our strategy provides a feasible and rational way to guide the zeolite synthesis field in generating novel frameworks. However, certain pre-requirements are needed – the incorporation of weakly bonded atoms. We revised the manuscript to emphasis the pre-requirements as given below.

The following text on page 1,

“This method opens a new way for synthesis of targeted zeolites, ...”, has been revised to

“This method provides a new concept for the synthesis of targeted zeolites, ...”

The following text on page 3,

“By adopting this approach, a series of targeted hypothetical zeolite structures are predicted,

and these structures are synthetically feasible.”, has been revised to

“By adopting this approach, we have, in addition, predicted a series of targeted hypothetical

zeolite structures.”

The following discussions have been added on page 9,

“We have demonstrated that many hypothetical zeolites can be predicted from known zeolite frameworks based on the *dcc*-*nsc* transformation. The 3D-3D topotactic transformation would provide a simple approach to generate novel zeolite frameworks, which may be inaccessible by conventional hydrothermal/solvothermal synthesis. We have also shown by molecular dynamics simulations that the weakened bonding, such as penta- or hexa-coordinated Al atoms, is crucial to initiate the 3D-3D topotactic transformation. Therefore, to synthesize new structures by this approach, it is important to introduce penta- or hexa-coordinated Al atoms in *dcc*-containing zeolite frameworks, and additional exploratory work is needed. Previous studies showed that certain types of OSDAs, like easily protonatable amines, could promote the formation of bridging Al-OH-Al bonds in AlPO4 molecular sieves, and penta- or hexa-coordinated Al atoms have been found in c.a. 25% of these materials, including AlPO4-17, AlPO4-41, AlPO4-EN3, AlPO-CJB1, VPI-5, IST-1, PST-13, etc. ^41,44–50^ The 3D-3D topotactic transformation may also be applicable to other zeolite compositions. For example, the gallophosphate Mu-18 (UEI) contains both *dcc* and penta-coordinated atoms (Fig. 4a6)^51^. We anticipate that with the discovery of many new zeolites, the approach described here will be more feasible.”

6

This is a very nice study of the transformation of a structurally hydrated AlPO4 phase upon calcination and certainly of high interest for researchers interested in formation and structure of porous materials.

**Reviewer #2**

The solved structure is quite convincing and consistent with observation.

***Response***: We are encouraged by the reviewer, who considers our work highly interesting. We also appreciate the reviewer’s valuable suggestions that encouraged us to improve the scientific quality of manuscript. Please find our detailed responses to the comments.

However, it is not conclusively demonstrated that the resulting phase (PST-6) fully corresponds to the already published PSI framework: a thorough structure analysis of the XRD-pattern obtained after calcination could not be found.

***Response***: A thorough structure analysis of the calcined PST-6 has been conducted and reported in *Angew. Chem. Int. Ed.* 53, 7480 (2014). The Rietveld refinement is shown in Figure R3.

**Figure R3.** Rietveld refinement for dehydrated PST-6: observed data (crosses), calculated fit (solid line), and difference plot (lower trace). Tick marks show the positions of allowed reflections (λ = 0.825621 Å). From *Angew. Chem. Int. Ed. 53, 7480 (2014).*

We compared the calcined phase, dehydrated PST-6, to the simulated PXRD pattern of PSI. The simulated and observed patterns agree well with each other. We added a new Figure S11 to the Supporting Information, and revised the following text on page 4,

“*In situ* powder XRD shows that the structural transformation of PST-5 to PST-6 starts at 200 ºC, and finishes at 500 ºC (Fig. S10)” to

“*In situ* PXRD shows that the structural transformation of PST-5 to PST-6 starts at 200 ºC, and finishes at 500 ºC (Fig. S10), which is accompanied by notable changes in the PXRD pattern. The observed pattern after calcination of PST-5 at 500 ºC agrees well with the simulated pattern from the PST-6 model, confirming the calcined PST-5 (i.e. PST-6) has the PSI framework (Fig. S11).”

7

**Figure S11.** (a) *In-situ* PXRD patterns of dehydrated PST-6 after heating PST-5 under vacuum at 800 °C. (b) PXRD pattern simulated from the PSI structure. Peaks from minor impurity phase and from Pt sample holder are marked by asterisks.

Furthermore, it is difficult to assess if the resulting phase is a fully ordered material as the X-ray data are presented in low graphical resolution and shows – as far as can be discerned – linebroadening. Linebroadening also is obvious from the NMR data, where the pattern of the calcined phase appears as a broadened, but very similar pattern compared to PST-5. To exclude the formation of a disordered framework, ED would be necessary, to clearly demonstrate no steaking occurs along any axis. Interestingly, the previously published work on PST-6 seems to indicate this material indeed is displaying stacking faults. The published ED data had shown streaking, even though this was not addressed by the authors previously.

***Response***: The resulting phase PST-6 indeed contains defects, as mentioned in our previous publication (*Angew. Chem. Int. Ed. 53, 7480,* **2014**). On the contrary, we have not observed any indications of disorder for PST-5. As shown in the 3D reciprocal lattices reconstructed from the (c)RED data in Figure R4, well-separated sharp spots are found for PST-5, while for PST-6, there are streaks along *a** showing the presence of disorder.

**Figure R4.** 3D reconstructed reciprocal lattice viewed along [010] of (a) PST-5 and (b) PST-6.

8

To clarify the disorder in PST-6 structure, we have changed the following sentence on page 6, “This orientation change promotes the disorder in PST-6^35^.” to

“The resulting PST-6 (PSI) material contains stacking faults, as indicated by the presence of streaks in the electron diffraction patterns.^35^”

Nonetheless, the work has merit, because this is a further and quite convincing example on the transformation of a porous structure containing 5-fold coordinated Al sites upon deep thermal dehydration. On the other hand, this observation is not novel and commonly observed in AlPO4 phases. The simultaneous occurrence of local inversion and resulting restructuring likewise is no real surprise.

***Response***: We agree with the reviewer that the 3D-3D transformation of a structure containing 5-coordinated Al sites upon deep thermal dehydration is no real surprise. However, this has not been identified as a general approach to targeted synthesis of novel zeolites. In this manuscript, we have for the first time studied the common features in the transformation of three aluminophosphates, and proposed a new concept for generating novel and synthetically feasible zeolites via 3D-3D topotactic transformations.

The purely theoretical enumeration of hypothetical frameworks which might result from a systematic phase transformation based on the deduced layers is quite nice and appealing in a topological view, but unless a clear strategy is suggested on how a deep dehydration can be directed to one specific and pure framework it remains a very intriguing brain-game only.

***Response***: We thank the reviewer for bringing this point to our attention. We have now performed more detailed investigations on the transformations of the three existing examples AlPO4-C to AlPO4-D, AlPO4-21 to AlPO4-25, and PST-5 to PST-6, and identified both their common and their unique features in the transformations. By applying similar transformations observed in the three known zeolites to other zeolites in the database, we generated more than 12 hypothetical zeolite structures that are synthetically feasible. In addition, we found the number of zeolite structures that can be generated from this approach is enormous. We also proposed possible strategies for the realization of the targeted zeolites.

In the revised manuscript, the last part of the main text related to the development of the new concept for targeted synthesis of novel zeolites has been completely revised, see page 7-10 including Figure 4 and Table 1. Accordingly we also revised the text in the section Hypothetical Zeolite Structure Generation on page S6-7 as well as Figures S14-18 and Tables S4-6 in the Supplementary Information. We have removed Figure 5 and related discussions on hypothetical *dcc*-containing zeolites generated from known *nsc-*containing zeolites, in order to focus on the already observed *dcc* to *nsc* transformations.

In summary: This contribution has many strong points, is pleasant to read but has a number of shortcomings, which leads this reviewer to advise its rejection for Nature Communications. However, for a specialized readership the article’s content certainly is of high interest.

***Response***: Thanks to the reviewer’s comments and suggestions. The novelties of our manuscript are not only the structural analysis of PST-5 and the discovery of the transformation of PST-5 to PST-6 via d*cc*/*d*4*r* to *nsc*. More importantly we proposed for the first time a new concept to targeted synthesis of novel zeolites in the aluminophosphate system, by 3D-3D topotactic

9

transformation. Based on the analyses of transformations in the three existing examples AlPO4-C to AlPO4-D, AlPO4-21 to AlPO4-25, and PST-5 to PST-6, we believe the concept is general and can be applied for synthesis of targeted zeolites, especially those that may not be feasible by conversional methods. We hope the revised manuscript has addressed the comments raised by the reviewer.

**Reviewer #3**

This is a really interesting article. The ADOR method was the first to show a completely new solid-solid transition mechanism for zeolite synthesis. This paper presents a second. I have only a few comments.

***Response***: We appreciate the reviewer to point out the novelty and significance of our work. We also thank the reviewer for the valuable suggestions for the improvement of the manuscript, which we have addressed in the revised version.

1) These two sentences do not appear to be logical: "In AlPO4 frameworks, the AlO4 and PO4 tetrahedra always alternate according to Löwenstein's rule32. Nevertheless, a pair of AlO4tetrahedra can be bridged by a hydroxyl group to adopt a trigonal bipyramidal-coordination, as found in AlPO4-21 (AWO)33 and AlPO4-C (APC)3" The authors need to say something like "when pentavalent Al is allowed" after "Nevertheless" to make these logical.

***Response***: We thank the reviewer for the suggestion. The following text on pages 2 and 3,

“Nevertheless, a pair of AlO4 tetrahedra can be bridged by a hydroxyl group to adopt a trigonal bipyramidal-coordination, as found in AlPO4-21 (AWO)^33^ and AlPO4-C (APC)^34^”, has been revised to

“Nevertheless, Al can adopt penta-coordination with a pair of Al atoms bridged by a hydroxyl group as found in AlPO4-21 (AWO)^33^ and hexa-coordination with two additional water molecules, for example, in AlPO4-C (APC)^34^.”

2) There is some confusion the text whether the pentavalent Al in PST-5 imply that dcc or d4r chains necessarily contain pentavalent Al. This should be clarified.

***Response***: We thank the reviewer for the comment. In the revised version, we have added the following new paragraph on page 9 to clarify this point and also to address the points #4 and #7 raised by the reviewer.

“We have demonstrated that many hypothetical zeolites can be predicted from known zeolite frameworks based on the *dcc*-*nsc* transformation. The 3D-3D topotactic transformation would provide a simple approach to generate novel zeolite frameworks, which may be inaccessible by conventional hydrothermal/solvothermal synthesis. We have also shown by molecular dynamics simulations that the weakened bonding, such as penta- or hexa-coordinated Al atoms, is crucial to initiate the 3D-3D topotactic transformation. Therefore, to synthesize new structures by this approach, it is important to introduce penta- or hexa-coordinated Al atoms in *dcc*-containing zeolite frameworks, and additional exploratory work is needed. Previous studies showed that certain types of OSDAs, like easily protonatable amines, could promote the formation of bridging Al-OH-Al bonds in AlPO4 molecular sieves, and penta- or hexa-coordinated Al atoms have been

10

found in c.a. 25% of these materials, including AlPO4-17, AlPO4-41, AlPO4-EN3, AlPO-CJB1, VPI-5, IST-1, PST-13, etc. ^41,44–50^ The 3D-3D topotactic transformation may also be applicable to other zeolite compositions. For example, the gallophosphate Mu-18 (UEI) contains both *dcc* and penta-coordinated atoms (Fig. 4a6)^51^. We anticipate that with the discovery of many new zeolites, the approach described here will be more feasible. ”

3) This sentence "It should be noted that molecular dynamics simulation of a hypothetical “pure” AlPO4 PST-5 structure without penta-coordinated Al atoms and bridging OH groups showed no cleavage of any framework bonds over the same timescale at 500 oC" is confusing, because the text had implied PST-5 contains pentavalent Al. What the "pure" structure is should be clarified somewhere, may in the SI.

***Response***: We thank the reviewer for the comment. In the revised version, the following text in the original version (page 6),

“It should be noted that molecular dynamics simulation of a hypothetical “pure” AlPO4 PST-5 structure without penta-coordinated Al atoms and bridging OH groups showed no cleavage of any framework bonds over the same timescale at 500 ^o^C.”, has been changed to

“It should be noted that the molecular dynamics simulations of an idealised, fully four-connected, AlPO4 PST-5 framework, without penta-coordinated Al atoms and bridging OH groups, showed no cleavage of any framework bonds over the same timescale at 500 ^o^C.” (see page 7) .

4) Related to #2 and "This implies that new zeolite frameworks can be synthesized via exploitation of the dcc-nsctransformation" the issue of pentavalency needs to be clarified. Zeolites do not contain pentavalent T atoms.

***Response***: Please see our response to the comment #2.

5) It would be helpful in Figure 4,5 to have arrows pointing from parent to hypothetical transformed zeolites (as there are in Figure 3).

***Response***: We appreciate the reviewer’s suggestion. We revised Figure 4 and added more examples of hypothetical structures generated by replacing *dcc* by *nsc*. Arrows have been added to point from parent to hypothetical zeolites. We have removed Figure 5 and related discussions on hypothetical *dcc*-containing zeolites generated from known *nsc-*containing zeolites, in order to focus on the already observed *dcc* to *nsc* transformations.

6) I do not seem to understand Table 1. What does FTC stand for? Which is parent and which is transformed zeolite?

***Response***: FTC stands for framework type code, which is approved by the Structure Commission of International Zeolite Association. We have added now a notation in Table 1,

[a] Framework Type Code approved by International Zeolite Association.”

We have also revised the Table 1 and clarified the parent and transformed zeolites.

11

Also, how is the energy calculated (SLC or ab initio)?

***Response***: We calculated the framework energies of hypothetical zeolite models relative to α-quartz using the SLC potential in the GULP program. The following description can be found in the “Hypothetical Zeolite Structure Generation” part of the Supplementary Information (page S6).

“To evaluate the feasibility of hypothetical models with *P*1 symmetry, their framework energies relative to α-quartz^31^ were calculated using the SLC potential in the GULP program. In addition, the local interatomic distances (LID) criteria were applied.^32^”

7) As can be seen from my comments, I am unclear about the transformation mechanism for known zeolites. Are the authors saying known zeolite 1 can become pentavalent and then can transform back to four-valent known zeolite 2? So, the intermediate state only is pentavalent? And this would occur only with Al-containing zeolites? This should all be explicitly stated.

***Response***: The reviewer’s understanding about the transformation is correct. We have addressed all those questions in the revised manuscript, see our response to the comment #2.

**Below are the list of additional changes we made in the revised manuscript**

1. Table S6 with the average bond lengths has been removed. The relevant information is already given in the text on page 5

“The key to the chain transformation is the penta-coordinated Al atoms, where Al-O bond lengths (on average 1.83(9) Å) are longer than those of tetra-coordinated Al atoms (on average 1.72(1) Å). “

2. In order to focus more on the generation of new zeolite structures from *dcc* to *nsc* transformation, we removed Figure 5 that presents hypothetical structures generated by replacing *nsc* with *dcc* or *d*4*r* and related text in the manuscript.
3. We have added the reflection list and refinement details in the PST-5_ED.cif file (CCDC database with deposition number 1944918).
4. All the hypothetical structures are included in a cif file (Hypothetical_Structures.cif) and added as a new Supplementary Information.
5. We have further improved the language.

**Reviewer comments, second version:**

REVIEWERS' COMMENTS:

Reviewer #1 (Remarks to the Author):

In the revised manuscript, the concerns I raised in the first round of review have been addressed.

Yu Han


Reviewer #2 (Remarks to the Author):

The manuscript has greatly been improved and the reasoning of the authors is much clearer now. Nonetheless, and despite the high quality of the scientific methodology, this reviewer is not convinced the topic is interesting to a large community, asides scientists in the field of porous materials, specifically tetrahedral frameworks. For this reason, it is suggested to publish the work in a more specialised journal.


Reviewer #3 (Remarks to the Author):

This is interesting work. The authors have responded to my comments.

**Author rebuttal, second version:**

Reviewer #1 (Remarks to the Author):

In the revised manuscript, the concerns I raised in the first round of review have been addressed.

Yu Han

Reviewer #2 (Remarks to the Author):

The manuscript has greatly been improved and the reasoning of the authors is much clearer now. Nonetheless, and despite the high quality of the scientific methodology, this reviewer is not convinced the topic is interesting to a large community, asides scientists in the field of porous materials, specifically tetrahedral frameworks. For this reason, it is suggested to publish the work in a more specialised journal.

Reviewer #3 (Remarks to the Author):

This is interesting work. The authors have responded to my comments.

**Reviewer comments, third version:**

**Author rebuttal, third version:**
